# Supplementary material for: Shared inflammatory glial cell signature after stab wound injury, revealed by spatial, temporal, and cell-type-specific profiling of the murine cerebral cortex
Source: Nat Commun. 2024 Apr 3;15:2866. doi: 10.1038/s41467-024-46625-w (PMC10991294; doi:10.1038/s41467-024-46625-w)
Supplement: Supplementary file 1 — Supplementary Information [file 41467_2024_46625_MOESM1_ESM.pdf]

Supplementary Information File

Shared inflammatory glial cell signature after stab wound injury, revealed by spatial, temporal, and cell-type-specific profiling of the murine cerebral cortex

Christina Koupourtidou[1,2,14], Veronika Schwarz[1,2,14], Hananeh Aliee[3], Simon Frerich[2,4], Judith Fischer-Sternjak[5,6], Riccardo Bocchi[5,6,7], Tatiana Simon-Ebert[5,6], Xianshu Bai[8,9], Swetlana Sirko[5,6], Frank Kirchhoff[8,9,10], Martin Dichgans[4,11],[12], Magdalena Götz[5,6,11], Fabian J. Theis[3,13], Jovica Ninkovic[1,6,11*]

[1]Chair of Cell Biology and Anatomy, Biomedical Center (BMC), Faculty of Medicine, LMU Munich, Planegg-Martinsried, Germany
[2]Graduate School of Systemic Neurosciences, LMU Munich, Munich, Germany
[3]Institute of Computational Biology, Helmholtz Zentrum München-German Research Center for Environmental Health, Neuherberg, Germany
[4]Institute for Stroke and Dementia Research, University Hospital, LMU Munich, Munich, Germany
[5]Chair of Physiological Genomics, Biomedical Center (BMC), Faculty of Medicine, LMU Munich, Planegg-Martinsried, Germany
[6]Institute of Stem Cell Research, Helmholtz Zentrum München-German Research Center for Environmental Health, Neuherberg, Germany
[7]Department of Basic Neurosciences, University of Geneva, Geneva, Switzerland
[8]Molecular Physiology, Center for Integrative Physiology and Molecular Medicine, University of Saarland, Homburg, Germany
[9]Center for Gender-specific Biology and Medicine (CGBM), University of Saarland, Homburg, Germany
[10]Experimental Research Center for Normal and Pathological Aging, University of Medicine and Pharmacy of Craiova, 200349 Craiova, Romania
[11]Munich Cluster for Systems Neurology SYNERGY, LMU Munich, Munich, Germany
[12]German Centre for Neurodegenerative Diseases, Munich, Germany
[13]Department of Mathematics, Technical University of Munich, Munich, Germany
[14]These authors contributed equally: Christina Koupourtidou, Veronika Schwarz
[*]Correspondence to: jovica.ninkovic@helmholtz-munich.de

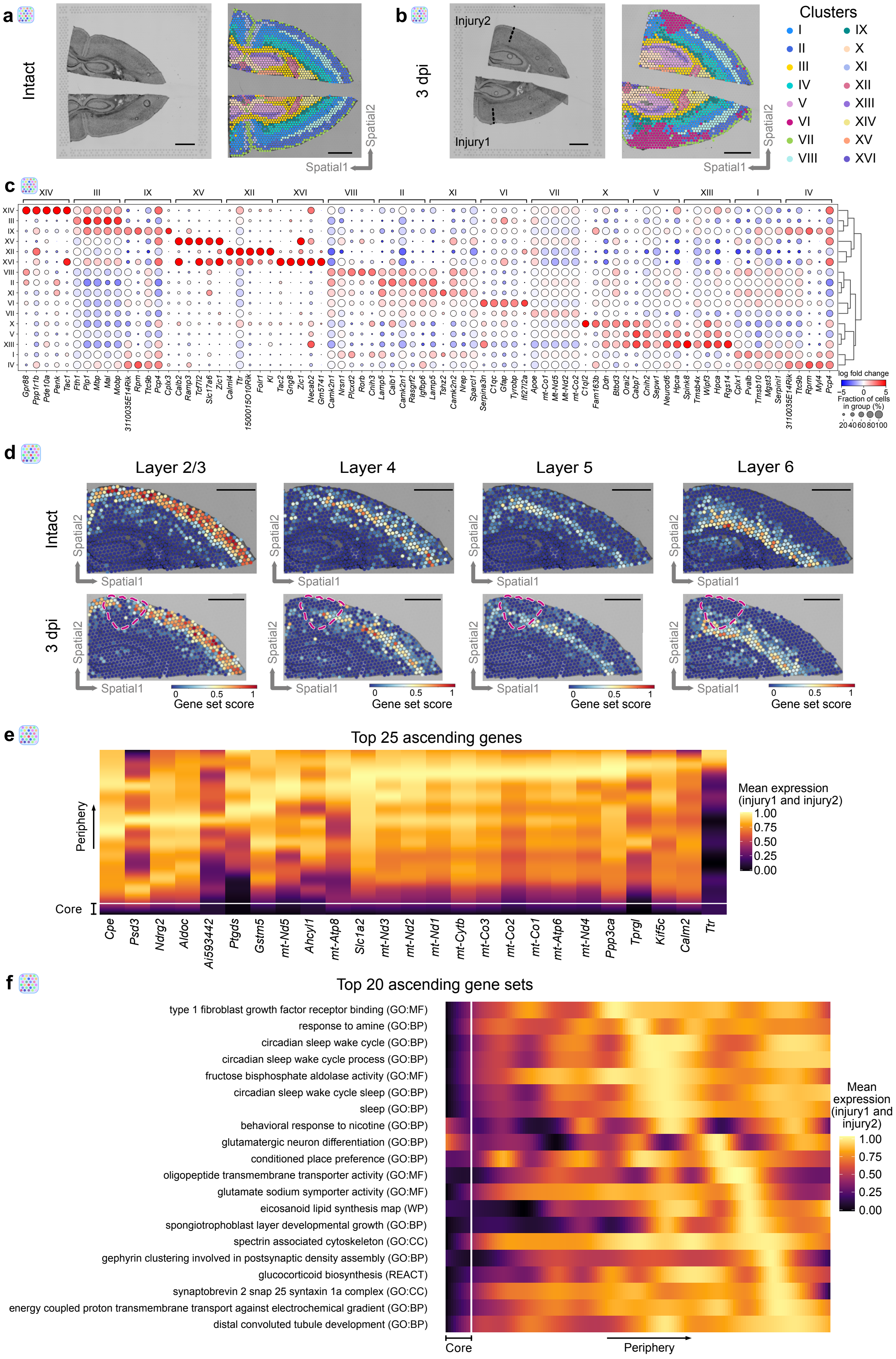

a
Intact
Spatial2
Spatial1
b
3 dpi
Injury2
Injury1
Spatial2
Spatial1
Clusters
I
II
III
IV
V
VI
VII
VIII
IX
X
XI
XII
XIII
XIV
XV
XVI
c
XIV III IX XV XII XVI VIII II XI VI VII X V XIII I IV
Gpr88 Ppp1r1b Pde10a Penk Tac1 Fth1 Plp1 Mbp Mal Mobp 3110035E14Rik Rpm Ttc9b Pcp4 Cplx3 Calb2 Ramp3 Tcf7l2 Slc17a6 Zic1 Calml4 Ttr 1500015O10Rik Folr1 Kl Tac2 Gng8 Zic1 Necab2 Gm5741 Camk2n1 Nrsn1 Plcxd2 Rorb Cnih3 Lamp5 Calb1 Camk2n1 Rasgrf2 Igfbp6 Lamp5 Tshz2 Camk2n2 Nrep Sparcl1 Serpina3n C1qc Gfap Tyrobp Ifi27l2a Apoe mt-Co1 Mt-Nd5 Mt-Nd2 mt-Co2 C1ql2 Fam163b Ddn Btbd3 Orai2 Cabp7 Cnih2 Sepw1 Neurod6 Hpca Spink8 Tmsb4x Wipf3 Hpca Rgs14 Cplx1 Pvalb Tmsb10 Mgst3 Serpini1 3110035E14Rik Ttc9b Rprm Myl4 Pcp4
log fold change
-5 0 5
Fraction of cells in group (%)
20 40 60 80 100
d
Layer 2/3
Layer 4
Layer 5
Layer 6
Intact
3 dpi
Spatial2
Spatial1
0 0.5 1
Gene set score
e
Top 25 ascending genes
Periphery
Core
Cpe Psd3 Ndrg2 Aldoc Ai593442 Ptgds Gstm5 mt-Nd5 Ahcyl1 mt-Atp8 Slc1a2 mt-Nd3 mt-Nd2 mt-Nd1 mt-Cytb mt-Co3 mt-Co2 mt-Co1 mt-Atp6 mt-Nd4 Ppp3ca Tprgl Kif5c Calm2 Ttr
Mean expression (injury1 and injury2)
1.00
0.75
0.50
0.25
0.00
f
Top 20 ascending gene sets
type 1 fibroblast growth factor receptor binding (GO:MF)
response to amine (GO:BP)
circadian sleep wake cycle (GO:BP)
circadian sleep wake cycle process (GO:BP)
fructose bisphosphate aldolase activity (GO:MF)
circadian sleep wake cycle sleep (GO:BP)
sleep (GO:BP)
behavioral response to nicotine (GO:BP)
glutamatergic neuron differentiation (GO:BP)
conditioned place preference (GO:BP)
oligopeptide transmembrane transporter activity (GO:MF)
glutamate sodium symporter activity (GO:MF)
eicosanoid lipid synthesis map (WP)
spongiotrophoblast layer developmental growth (GO:BP)
spectrin associated cytoskeleton (GO:CC)
gephyrin clustering involved in postsynaptic density assembly (GO:BP)
glucocorticoid biosynthesis (REACT)
synaptobrevin 2 snap 25 syntaxin 1a complex (GO:CC)
energy coupled proton transmembrane transport against electrochemical gradient (GO:BP)
distal convoluted tubule development (GO:BP)
Core
Periphery
Mean expression (injury1 and injury2)
1.00
0.75
0.50
0.25
0.00

Supplementary Figure 1. Spatial transcriptome of intact and stab wound-injured mice.

a-**b** Spatial transcriptomics in intact and stab wound-injured mice (3 dpi). Brains were manually resected and positioned on 10x Visium capture areas. In each capture area, two brain sections of either intact (**a**) or stab wound-injured cortices (**b**) were collected. Clustering of gene expression data on spatial coordinates is based on highly variable genes and subsequent dimensionality reduction in two replicates. The dashed black lines indicate the injury. **c** Dot plot depicting the 5 most enriched genes in each of the 16 identified clusters (Supplementary Table 1). **d** Expression of neuronal layer scores 2/3, 4, 5 and 6 in intact and injured brain sections on spatial coordinates. The dashed pink lines highlight cluster VI borders. Neuronal layer gene set scores are listed in Supplementary Table 2. **e** Heatmap showing the expression of the 25 most ascending genes along the spatial trajectory (see Fig. 2) depicted as mean expression of injury 1 and 2. **f** Heatmap displaying the 20 most enriched ascending gene sets along the spatial trajectory (see Fig. 2) depicted as mean expression of injury 1 and 2. All data shown in this figure are based on n=1 animal per condition over 1 experiment, with two sections being captured. Injury 1 and 2 represent the injuries of each hemisphere in the same animal. Scale bars: **a**,**b**,**d**: 1 mm. Abbreviations: BP = biological processes, MF = molecular functions, CC = cellular components, GO = gene ontology, REACT = reactome, WP = WikiPathways, dpi = days post injury.

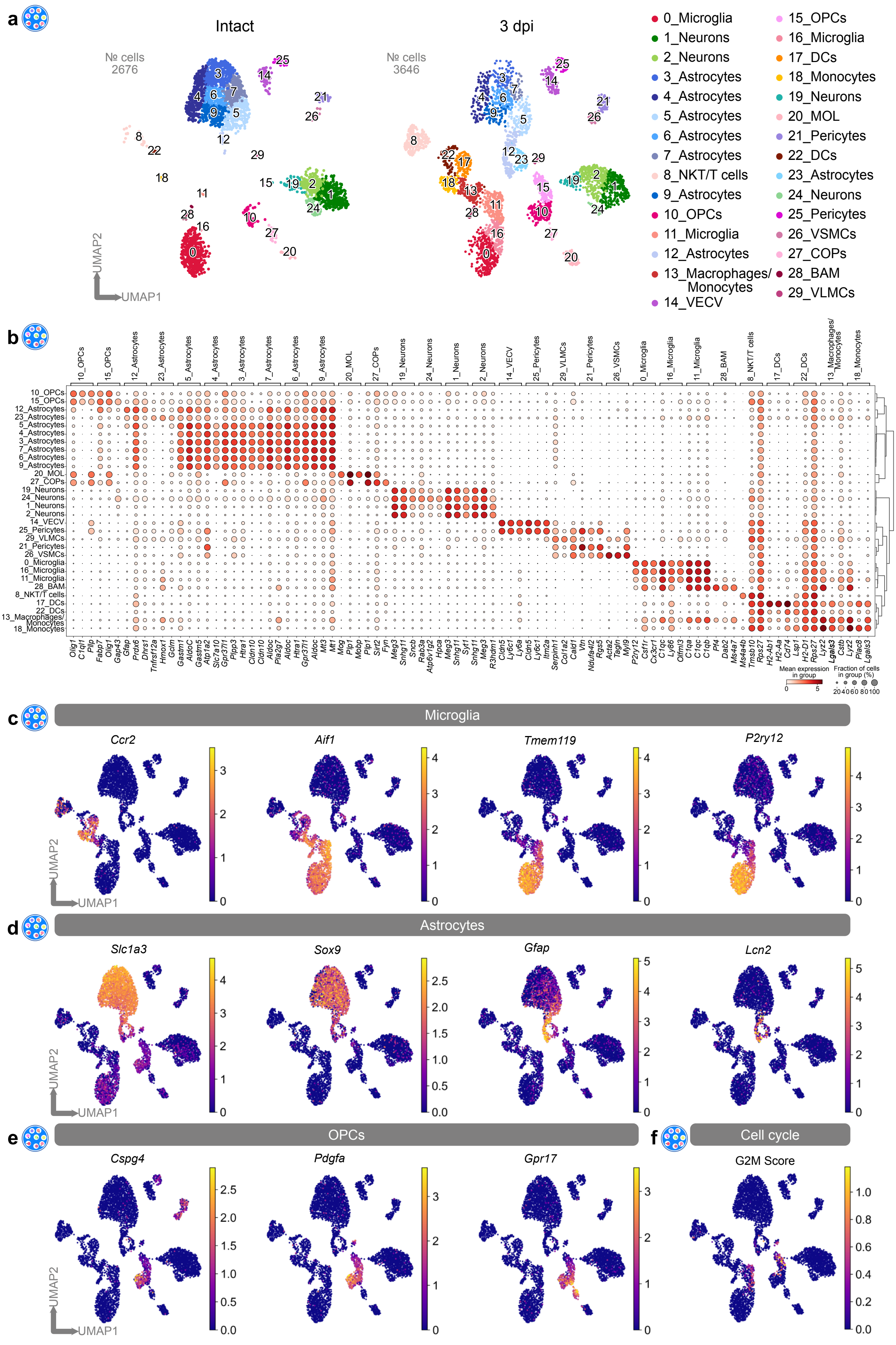
a
Intact
3 dpi
№ cells 2676
№ cells 3646
UMAP1
UMAP2
0_Microglia
1_Neurons
2_Neurons
3_Astrocytes
4_Astrocytes
5_Astrocytes
6_Astrocytes
7_Astrocytes
8_NKT/T cells
9_Astrocytes
10_OPCs
11_Microglia
12_Astrocytes
13_Macrophages/ Monocytes
14_VECV
15_OPCs
16_Microglia
17_DCs
18_Monocytes
19_Neurons
20_MOL
21_Pericytes
22_DCs
23_Astrocytes
24_Neurons
25_Pericytes
26_VSMCs
27_COPs
28_BAM
29_VLMCs
b
Mean expression in group
Fraction of cells in group (%)
c
Microglia
Ccr2
Aif1
Tmem119
P2ry12
d
Astrocytes
Slc1a3
Sox9
Gfap
Lcn2
e
OPCs
Cspg4
Pdgfa
Gpr17
f
Cell cycle
G2M Score

Supplementary Figure 2. scRNA-seq clustering of intact and stab wound-injured mice (3 dpi) and cell-type identification.

a UMAP plots depicting clustering of cells originating from intact (2676 cells) and injured (3646 cells) conditions among 30 defined clusters. Clusters are color-coded and annotated according to their transcriptional identities. Note that clusters 13_ Macrophages/Monocytes, 17_DCs, and 23_Astrocytes are almost absent in the intact condition. **b** Dot plot depicting the expression of the 3 most enriched genes in each of the 30 identified clusters (see Supplementary Data 3). **c**-**e** UMAPs highlighting example marker genes to identify microglia/macrophages (**c**), astrocytes (**d**) OPCs (**e**) and cycling cells (**f**). G2/M gene set score is listed in Supplementary Table 4. Data shown in this figure are derived from n=3 intact animals over 1 experiment (1 scRNAseq libray) and n=3 3dpi animals over 1 experiment (2 scRNAseq libraries). Abbreviations: UMAP = uniform manifold approximation and projection, dpi = days post injury, OPCs = oligodendrocyte progenitor cells, COPs = committed oligodendrocyte progenitors, MOL = mature oligodendrocytes, VECV = vascular endothelial cells (venous), VSMCs = vascular smooth muscle cells, VLMCs = vascular and leptomeningeal cells, BAM = border-associated macrophages, NKT cells = natural killer T cells, DCs = dendritic cells.

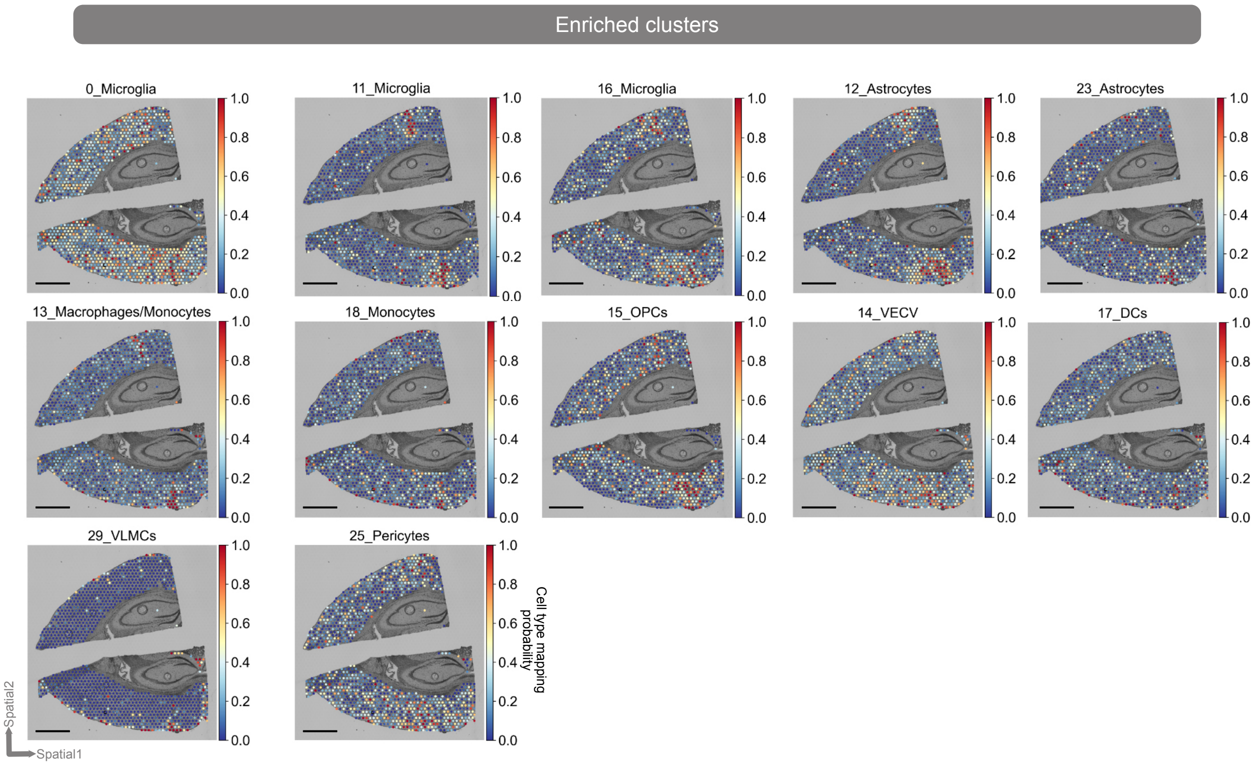
Enriched clusters
0_Microglia
11_Microglia
16_Microglia
12_Astrocytes
23_Astrocytes
13_Macrophages/Monocytes
18_Monocytes
15_OPCs
14_VECV
17_DCs
29_VLMCs
25_Pericytes
Cell type mapping probability
0.0
0.2
0.4
0.6
0.8
1.0
Spatial2
Spatial1

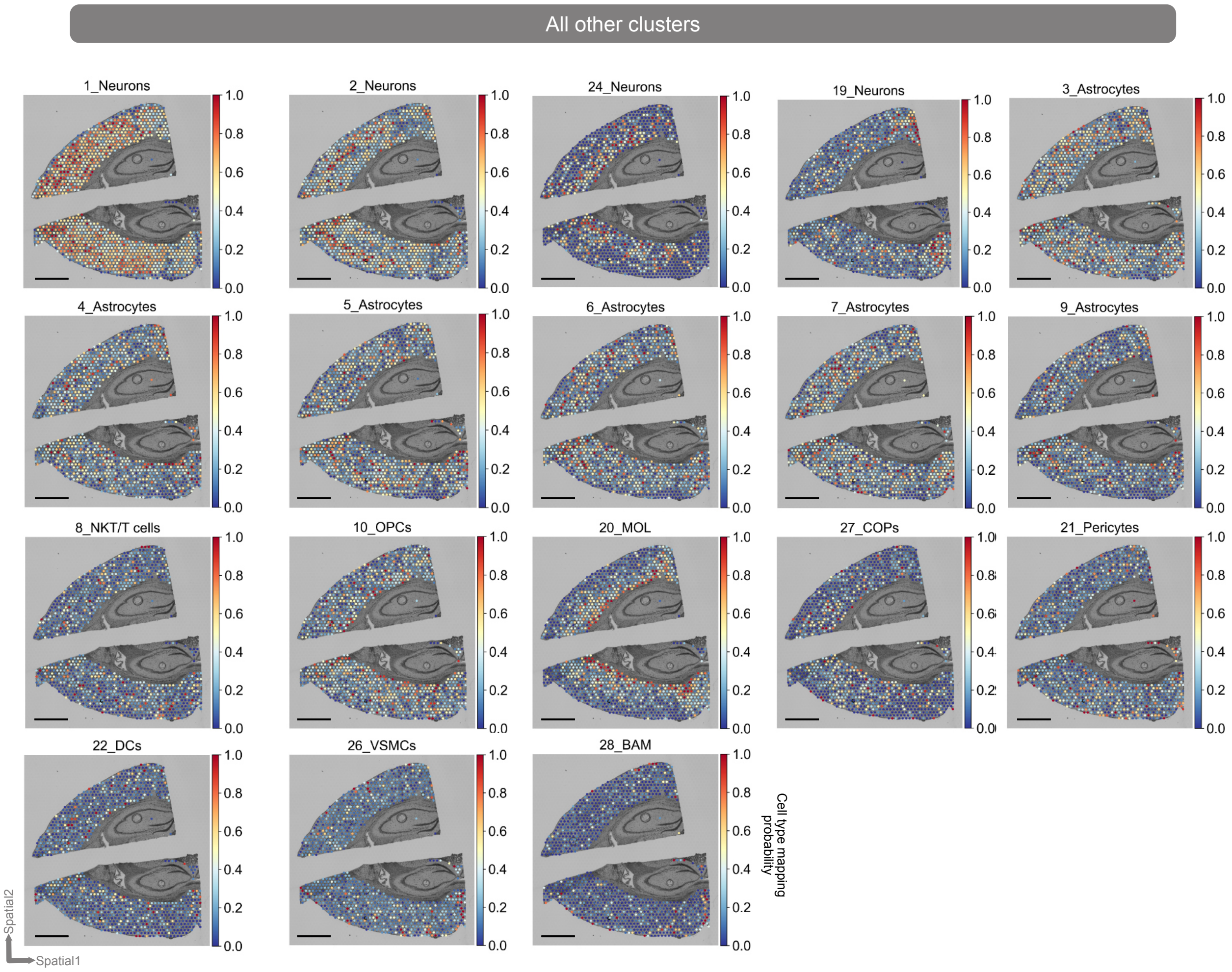
All other clusters
1_Neurons
2_Neurons
24_Neurons
19_Neurons
3_Astrocytes
4_Astrocytes
5_Astrocytes
6_Astrocytes
7_Astrocytes
9_Astrocytes
8_NKT/T cells
10_OPCs
20_MOL
27_COPs
21_Pericytes
22_DCs
26_VSMCs
28_BAM
Cell type mapping probability
0.0
0.2
0.4
0.6
0.8
1.0
Spatial2
Spatial1

Supplementary Figure 3. Probabilistic mapping of in scRNA-seq identified cellular clusters on Visium dataset.

Probabilistic mapping of in Fig. 3b identified scRNA-seq (3 dpi only) clusters on the spatial transcriptome dataset (3 dpi). Stab wound injury elicits different reaction modes in the injury vicinity. Plots are grouped into injury enriched clusters (upper panel) and all other clusters (lower panel). scRNAseq data shown in this figure are derived from n=3 intact animals over 1 experiment (1 scRNAseq libray) and n=3 3dpi animals over 1 experiment (2 scRNAseq libraries). stRNAseq data are based on n=1 animal over 1 experiment with two injured hemispheres being captured. Scale bars: 1 mm. Abbreviations: dpi = days post injury, OPCs = oligodendrocyte progenitor cells, NKT cells = natural killer T cells, DCs = dendritic cells, MOL = mature oligodendrocytes, VSMCs = vascular smooth muscle cells, COPs = committed oligodendrocyte progenitors, BAM = border-associated macrophages, VLMCs = vascular and leptomeningeal cells, VECV = vascular endothelial cells (venous).

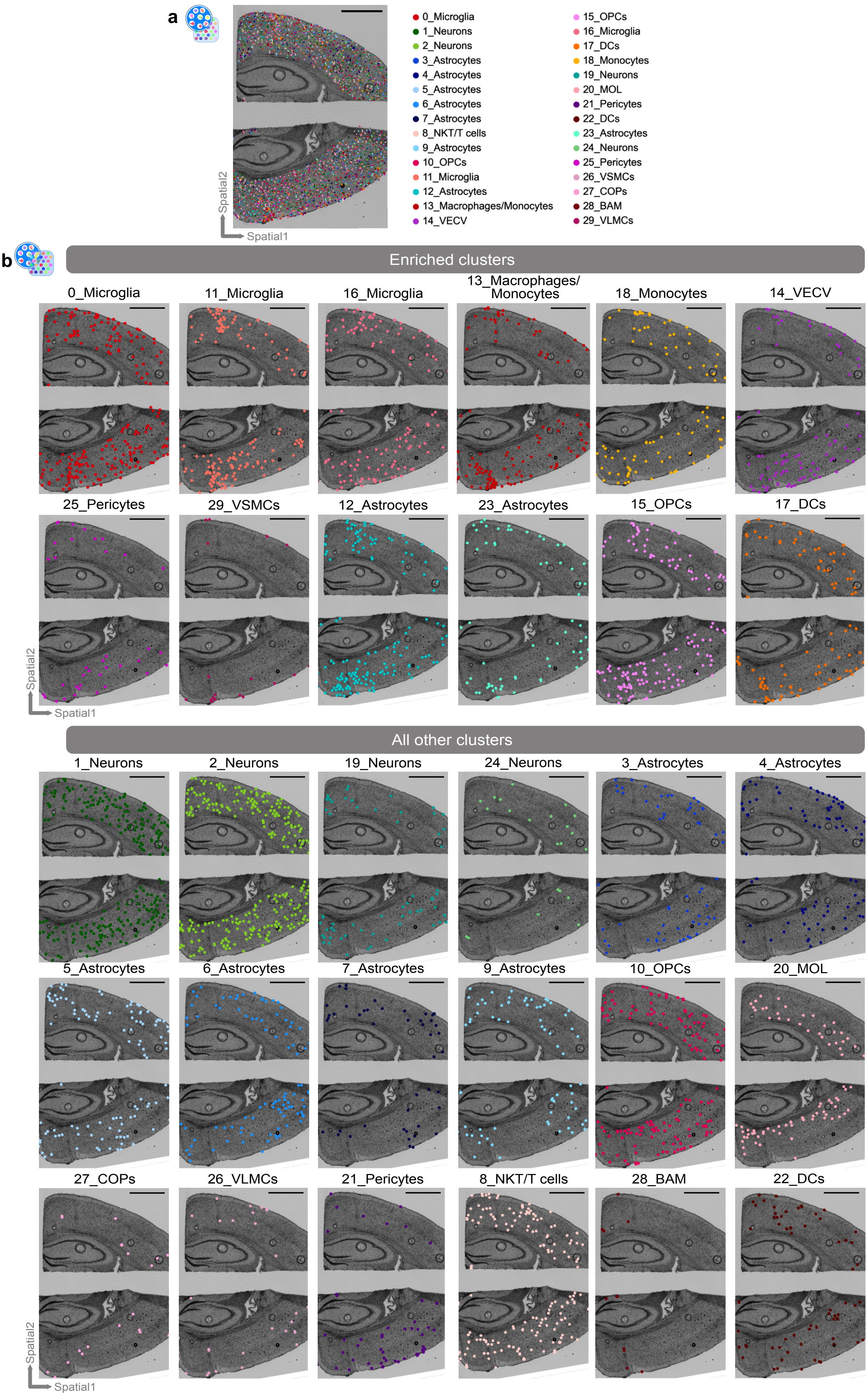
a
0_Microglia
1_Neurons
2_Neurons
3_Astrocytes
4_Astrocytes
5_Astrocytes
6_Astrocytes
7_Astrocytes
8_NKT/T cells
9_Astrocytes
10_OPCs
11_Microglia
12_Astrocytes
13_Macrophages/Monocytes
14_VECV
15_OPCs
16_Microglia
17_DCs
18_Monocytes
19_Neurons
20_MOL
21_Pericytes
22_DCs
23_Astrocytes
24_Neurons
25_Pericytes
26_VSMCs
27_COPs
28_BAM
29_VLMCs
Spatial2
Spatial1
b
Enriched clusters
0_Microglia
11_Microglia
16_Microglia
13_Macrophages/ Monocytes
18_Monocytes
14_VECV
25_Pericytes
29_VSMCs
12_Astrocytes
23_Astrocytes
15_OPCs
17_DCs
Spatial2
Spatial1
All other clusters
1_Neurons
2_Neurons
19_Neurons
24_Neurons
3_Astrocytes
4_Astrocytes
5_Astrocytes
6_Astrocytes
7_Astrocytes
9_Astrocytes
10_OPCs
20_MOL
27_COPs
26_VLMCs
21_Pericytes
8_NKT/T cells
28_BAM
22_DCs
Spatial2
Spatial1

Supplementary Figure 4. Single-cell deconvolution-based mapping of in scRNA-seq identified cellular clusters on Visium dataset.
a,**b** Deconvolution based mapping of in Fig. 3 identified scRNA-seq clusters on the spatial transcriptome dataset (3 dpi). Plots are grouped into injury enriched clusters (upper panel) and all other clusters (lower panel). scRNAseq data shown in this figure are derived from n=3 intact animals over 1 experiment (1 scRNAseq libray) and n=3 3dpi animals over 1 experiment (2 scRNAseq libraries). stRNAseq data are based on n=1 animal over 1 experiment with two injured hemispheres being captured. Scale bars: 1 mm. Abbreviations: dpi = days post injury, OPCs = oligodendrocyte progenitor cells, NKT cells = natural killer T cells, DCs = dendritic cells, MOL = mature oligodendrocytes, VSMCs = vascular smooth muscle cells, COPs = committed oligodendrocyte progenitors, BAM = border-associated macrophages, VLMCs = vascular and leptomeningeal cells, VECV = vascular endothelial cells (venous).

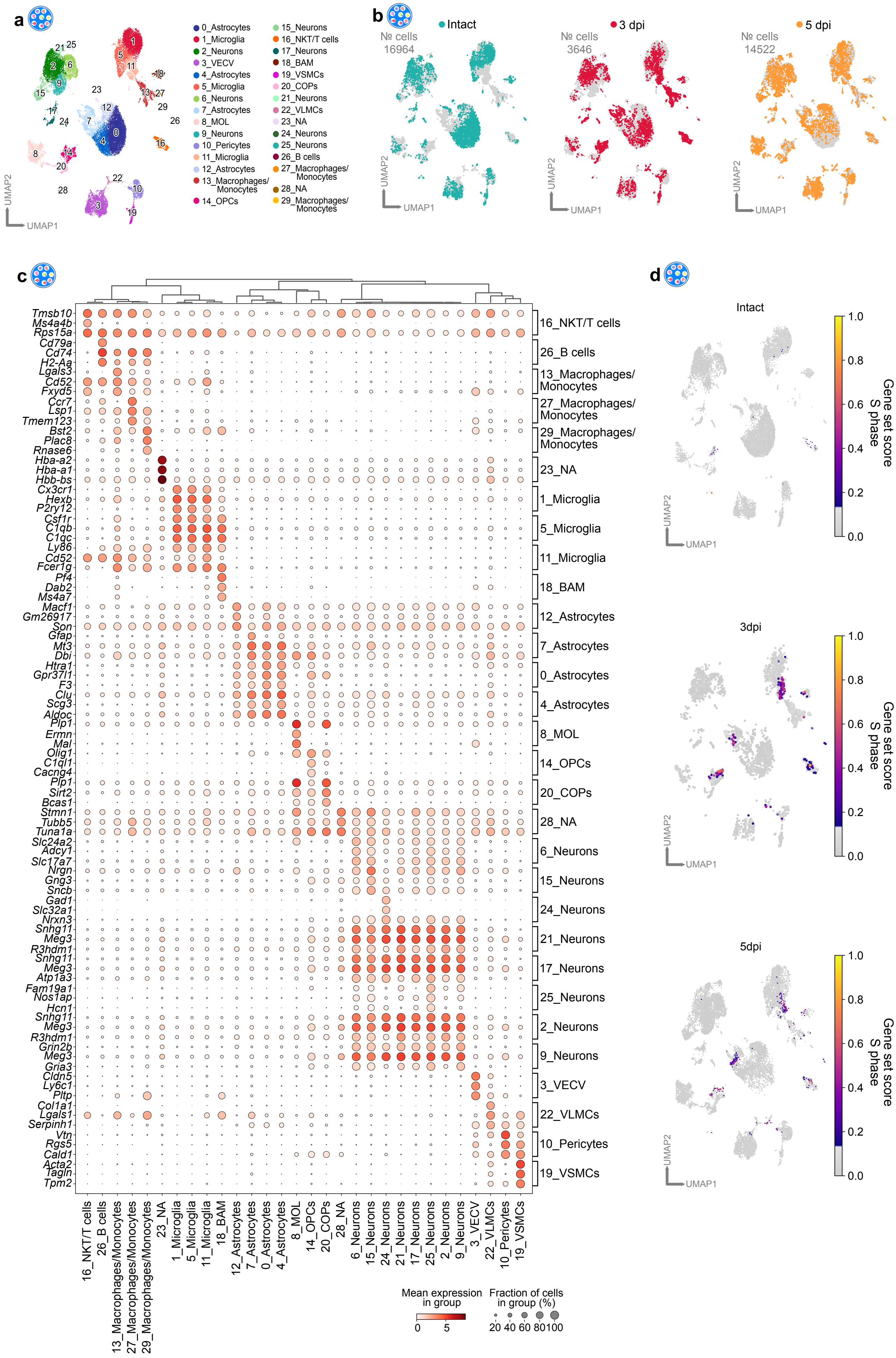
a
0_Astrocytes
1_Microglia
2_Neurons
3_VECV
4_Astrocytes
5_Microglia
6_Neurons
7_Astrocytes
8_MOL
9_Neurons
10_Pericytes
11_Microglia
12_Astrocytes
13_Macrophages/ Monocytes
14_OPCs
15_Neurons
16_NKT/T cells
17_Neurons
18_BAM
19_VSMCs
20_COPs
21_Neurons
22_VLMCs
23_NA
24_Neurons
25_Neurons
26_B cells
27_Macrophages/ Monocytes
28_NA
29_Macrophages/ Monocytes
UMAP1
UMAP2
b
Intact
№ cells 16964
3 dpi
№ cells 3646
5 dpi
№ cells 14522
c
d
Intact
3dpi
5dpi
Gene set score S phase
Mean expression in group
Fraction of cells in group (%)
20 40 60 80 100

Supplementary Figure 5. Integration of scRNA-seq datasets of intact and stab wound-injured mice (3 and 5 dpi), cluster distribution and cell type identification.

a,b UMAP plots depicting clustering (**a**) and cell distribution of intact (16964 cells), 3 dpi (3646 cells), and 5 dpi (14522 cells) cells among 30 defined clusters (**b**). Clusters were color-coded and annotated according to their transcriptional identities. **c** Dot plot depicting expression of the 3 most enriched genes in each of the 30 identified clusters (see Supplementary Data 4). **d** UMAP plots highlighting proliferating cells entering S-phase in intact and brain injured conditions (see Supplementary Table 4). Data shown in this figure are derived from n=12 intact animals over 3 independent experiments (5 scRNAseq libraries) , n=3 3dpi animals over 1 experiment (2 scRNAseq libraries), and n=9 5dpi animals over 3 independent experiments (3 scRNAseq libraries). Abbreviations: dpi = days post injury, UMAP = uniform manifold approximation and projection, dpi = days post injury, VECV = vascular endothelial cells (venous), MOL = mature oligodendrocytes, OPCs = oligodendrocyte progenitor cells, NKT cells = natural killer T cells, BAM = border-associated macrophages, VSMCs = vascular smooth muscle cells, COPs = committed oligodendrocyte progenitors, VLMCs = vascular and leptomeningeal cells, NA = not available.

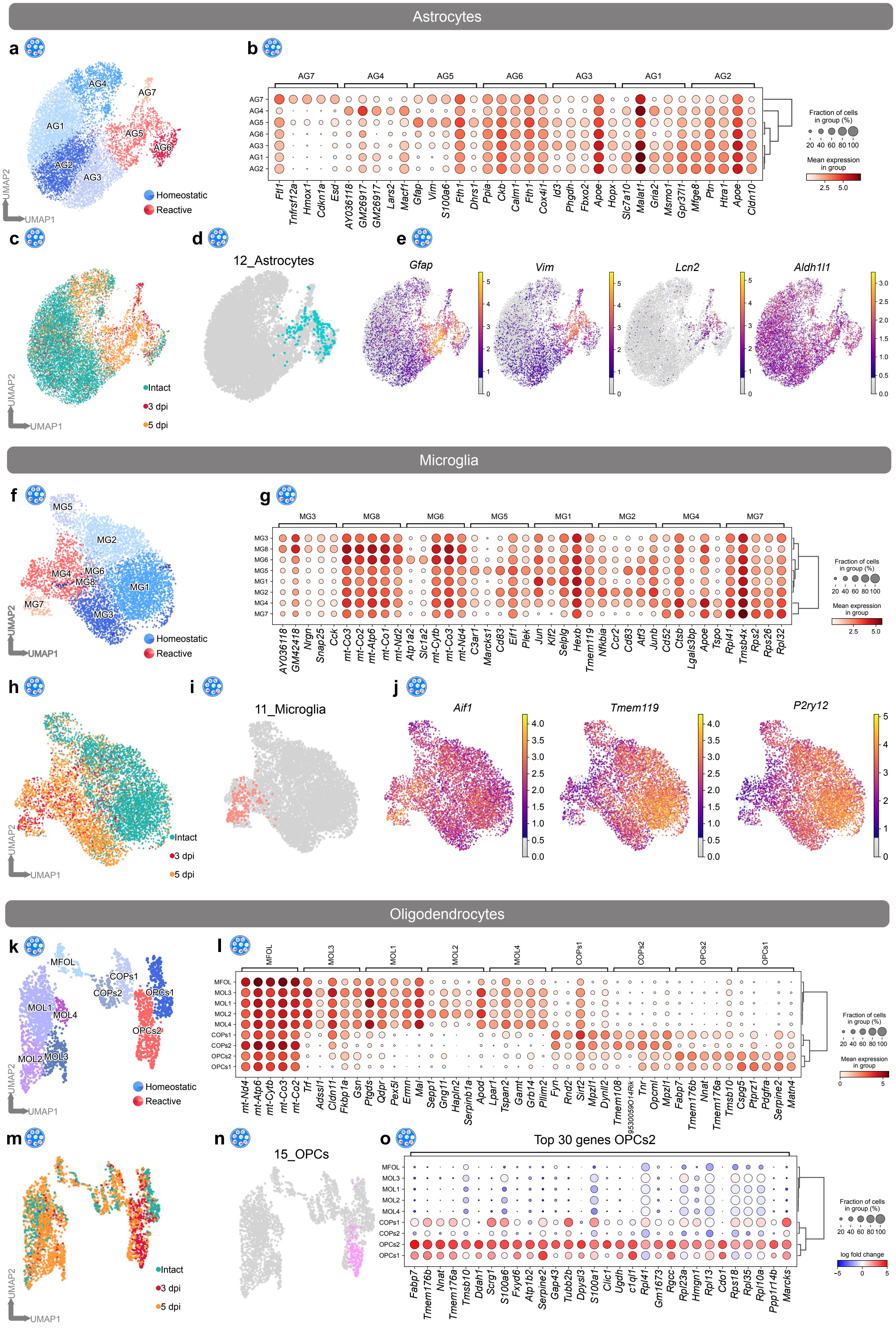
Astrocytes
a
AG4
AG7
AG1
AG5
AG6
AG2
AG3
Homeostatic
Reactive
UMAP2
UMAP1
b
AG7 AG4 AG5 AG6 AG3 AG1 AG2
Fraction of cells in group (%)
20 40 60 80 100
Mean expression in group
2.5 5.0
Ftl1 Tnfrsf12a Hmox1 Cdkn1a Esd AY036118 GM26917 GM26917 Lars2 Macf1 Gfap Vim S100a6 Fth1 Dhrs1 Ppia Ckb Calm1 Fth1 Cox4i1 Id3 Phgdh Fbxo2 Apoe Hopx Slc7a10 Malat1 Gria2 Msmo1 Gpr37l1 Mfge8 Ptn Htra1 Apoe Cldn10
c
Intact
3 dpi
5 dpi
d
12_Astrocytes
e
Gfap
Vim
Lcn2
Aldh1l1
Microglia
f
MG5
MG2
MG4
MG6
MG8
MG1
MG7
MG3
Homeostatic
Reactive
g
MG3 MG8 MG6 MG5 MG1 MG2 MG4 MG7
Fraction of cells in group (%)
Mean expression in group
AY036118 GM42418 Nrgn Snap25 Cck mt-Co3 mt-Co2 mt-Atp6 mt-Co1 mt-Nd2 Atp1a2 Slc1a2 mt-Cytb mt-Co3 mt-Nd4 C3ar1 Marcks1 Cd83 Eif1 Plek Jun Klf2 Selplg Hexb Tmem119 Nfkbia Ccr2 Cd83 Atf3 Junb Cd52 Ctsb Lgals3bp Apoe Tspo Rpl41 Tmsb4x Rps2 Rps26 Rpl32
h
Intact
3 dpi
5 dpi
i
11_Microglia
j
Aif1
Tmem119
P2ry12
Oligodendrocytes
k
MFOL
COPs1
COPs2
OPCs1
MOL1
MOL4
OPCs2
MOL2
MOL3
Homeostatic
Reactive
l
MFOL MOL3 MOL1 MOL2 MOL4 COPs1 COPs2 OPCs2 OPCs1
Fraction of cells in group (%)
Mean expression in group
mt-Nd4 mt-Atp6 mt-Cytb mt-Co3 mt-Co2 Trf Adssl1 Cldn11 Fkbp1a Gsn Ptgds Qdpr Pex5l Ermn Mal Sepp1 Gng11 Hapln2 Serpinb1a Apod Lpar1 Tspan2 Gamt Grb14 Pllim2 Fyn Rnd2 Sirt2 Mpzl1 Dynll2 Tmem108 9530059O14Rik Tnr Opcml Mpzl1 Fabp7 Tmem176b Nnat Tmem176a Tmsb10 Cspg5 Ptprz1 Pdgfra Serpine2 Matn4
m
Intact
3 dpi
5 dpi
n
15_OPCs
o
Top 30 genes OPCs2
Fraction of cells in group (%)
log fold change
Fabp7 Tmem176b Nnat Tmem176a Tmsb10 Ddah1 Scrg1 S100a6 Fxyd6 Atp1b2 Serpine2 Gap43 Tubb2b Dpysl3 S100a1 Clic1 Ugdh c1ql1 Rpl41 Gm1673 Rgcc Rpl23a Hmgn1 Rpl13 Cdo1 Rps18 Rpl35 Rpl10a Ppp1r14b Marcks

Supplementary Figure 6. Stab wound injury elicits unique gene expression in distinct glial subpopulations.

a UMAP depicting subclustered astrocytes of integrated and batch-corrected intact, 3 dpi, and 5 dpi datasets. **b** Dot plot depicting expression of the 5 most enriched genes in each of the 7 identified clusters. **c** UMAP displaying cell distribution of intact (green), 3 dpi (red) and 5 dpi (orange) cells among all 7 astrocytic clusters. **d** UMAP displaying localization of injury-enriched cluster 12_Astrocytes (turquoise, Supplementary Fig. 4b) on subclustered astrocytes. **e** UMAPs highlighting expression of example marker genes *Gfap*, *Vim*, *Lcn2* and *Aldh1l1* to identify reactive astrocyte clusters. **f** UMAP depicting subclustered microglia of integrated and batch- corrected intact, 3 dpi, and 5 dpi datasets. **g** Dot plot depicting expression of the 5 most enriched genes in each of the 8 identified clusters. **h** UMAP displaying cell distribution of intact (green), 3 dpi (red), and 5 dpi (orange) cells among all 8 microglial clusters. **i** UMAP displaying localization of injury-enriched cluster 11_Microglia (peach, Supplementary Fig. 4b) on subclustered microglia. **j** UMAPs highlighting expression of example marker genes *Aif1*, *Tmem119* and *P2ry12* to identify reactive microglial clusters. **k** UMAP depicting subclustered oligodendrocytes of integrated and batch-corrected intact, 3 dpi, and 5 dpi datasets. **l** Dot plot depicting expression of the 5 most enriched genes in each of the 9 identified clusters. **m** UMAP displaying cell distribution of intact (green), 3 dpi (red), and 5 dpi (orange) cells among all 9 clusters. **n** UMAP displaying localization of injury-enriched cluster 15_OPCs (pink, Supplementary Fig. 4b) on subclustered oligodendrocytes. **o** Dot plot depicting expression of the 30 most enriched genes in cluster OPCs2. Note comparable gene expression of clusters OPCs1 and OPCs2 prevents the identification of unique reactive OPC markers. Data shown in this figure are derived from n=12 intact animals over 3 independent experiments (5 scRNAseq libraries) , n=3 3dpi animals over 1 experiment (2 scRNAseq libraries), and n=9 5dpi animals over 3 independent experiments (3 scRNAseq libraries). Abbreviations: UMAP = uniform manifold approximation and projection, AG = astroglia, dpi = days post injury, MG = microglia, OPCs = oligodendrocyte progenitor cells, COPs = committed oligodendrocyte progenitors, MFOL = myelin-forming oligodendrocytes, MOL = mature oligodendrocytes.

a

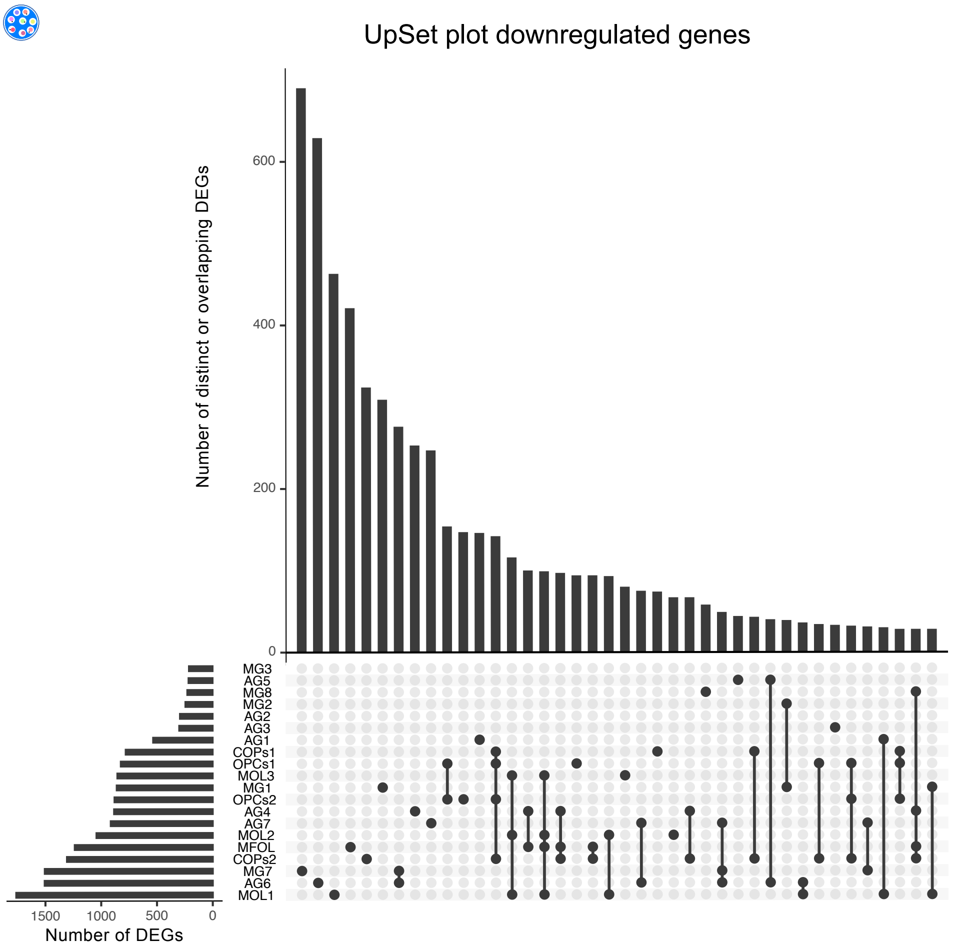
UpSet plot downregulated genes
Number of distinct or overlapping DEGs
0
200
400
600
MG3
AG5
MG8
MG2
AG2
AG3
AG1
COPs1
OPCs1
MOL3
MG1
OPCs2
AG4
AG7
MOL2
MFOL
COPs2
MG7
AG6
MOL1
1500
1000
500
0
Number of DEGs

b

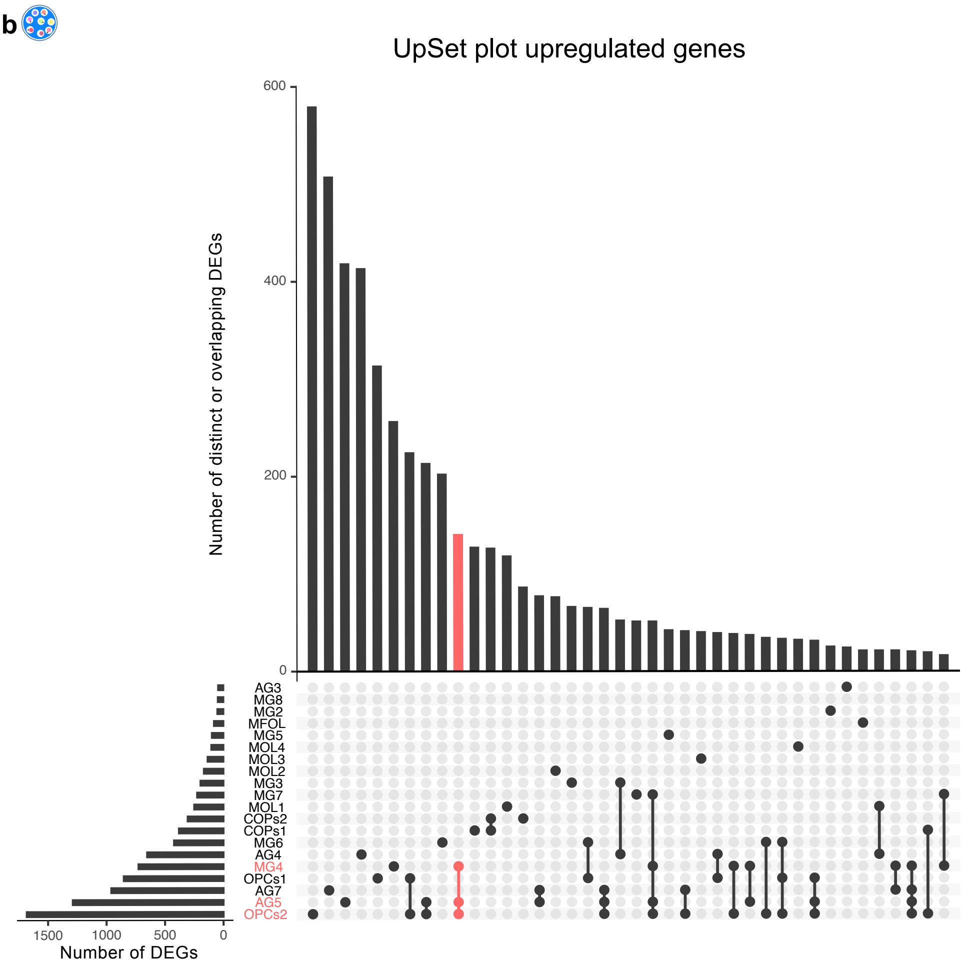
UpSet plot upregulated genes
Number of distinct or overlapping DEGs
0
200
400
600
AG3
MG8
MG2
MFOL
MG5
MOL4
MOL3
MOL2
MG3
MG7
MOL1
COPs2
COPs1
MG6
AG4
MG4
OPCs1
AG7
AG5
OPCs2
1500
1000
500
0
Number of DEGs

Supplementary Figure 7. Reactive glial subpopulations share injury-induced transcriptomic signature.

a,b UpSet plot illustrating downregulated (**a**) and upregulated (**b**) DEGs (pval < 0.05, $\log_2$ fold change > 1.6 or $\log_2$ fold change < -1.6, method: t-test overestimated_variance) across different glial subclusters of integrated and batch-corrected intact, 3 dpi, and 5 dpi datasets (Fig. 4c-e). Each horizontal bar represents number of DEGs within a specific cluster, and vertical bars indicate the number of unique or shared genes between clusters. Connected dots highlight shared DEGs across clusters, while single dots represent uniquely regulated DEGs. Wherever applicable, DEGs are determined by comparing each glial subcluster to all other subclusters within the respective cell type (further details in Methods). The red bar in (**b**) highlights commonly shared genes between reactive astrocytic, microglial, and oligodendroglial subclusters. Data shown in this figure are derived from n=12 intact animals over 3 independent experiments (5 scRNAseq libraries) , n=3 3dpi animals over 1 experiment (2 scRNAseq libraries), and n=9 5dpi animals over 3 independent experiments (3 scRNAseq libraries). Abbreviations: DEGs = differentially expressed genes, AG = astroglia, MG = microglia, OPCs = oligodendrocyte progenitor cells, COPs = committed oligodendrocyte progenitors, MFOL = myelin-forming oligodendrocytes, MOL = mature oligodendrocytes.

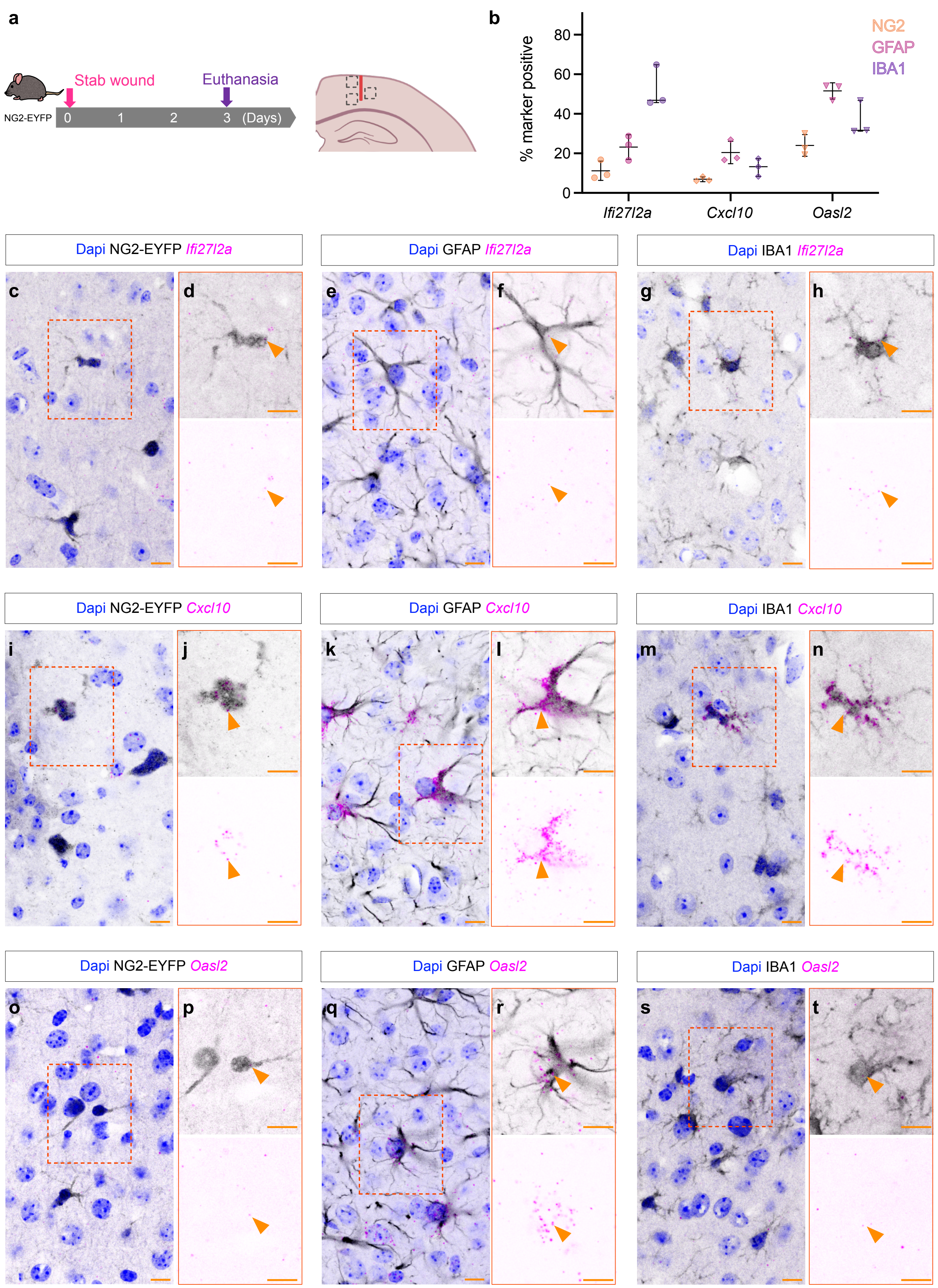
a
Stab wound
Euthanasia
NG2-EYFP
0 1 2 3 (Days)
b
% marker positive
80
60
40
20
0
Ifi27l2a
Cxcl10
Oasl2
NG2
GFAP
IBA1
Dapi NG2-EYFP Ifi27l2a
Dapi GFAP Ifi27l2a
Dapi IBA1 Ifi27l2a
c
d
e
f
g
h
Dapi NG2-EYFP Cxcl10
Dapi GFAP Cxcl10
Dapi IBA1 Cxcl10
i
j
k
l
m
n
Dapi NG2-EYFP Oasl2
Dapi GFAP Oasl2
Dapi IBA1 Oasl2
o
p
q
r
s
t

Supplementary Figure 8. Reactive glial subpopulations display shared gene expression following injury.

a Experimental paradigm for assessing shared gene expression in astrocytes, microglia, and OPCs (NG2-EYFP). The dashed gray boxes on the mouse brain scheme indicate the example imaging areas. The red line indicates the injury core. **b** Dot plot depicting the proportion of OPCs (NG2-EYFP, yellow), astrocytes (GFAP, pink) and microglia (IBA1, purple) expressing the selected shared innate immunity- associated genes *Ifi27l2a*, *Cxcl10*, and *Oasl2*. Data are shown as median ± 95% confidence interval. Each data point represents one animal (n = 3). Source data are provided as Source Data file. **c**-**t** Representative images of RNAscope for *Ifi27l2a* (**c**-**g**), *Cxcl10* (**i**-**m**), and *Oasl2* (**o**-**s**) (magenta) combined with GFP (NG2 glia) (black) (**c**,**i**,**o**), GFAP (black) (**e**,**k**,**q**), IBA1 (black) (**g**,**m**,**s**) immunostaining to identify cell types. Framed micrographs are magnifications of the red boxed areas in corresponding overview images. The orange arrowheads in micrograph depict double positive cells. All images are single z-plane of confocal z-stacks. Scale bars: **c**-**t**: 10 μm.

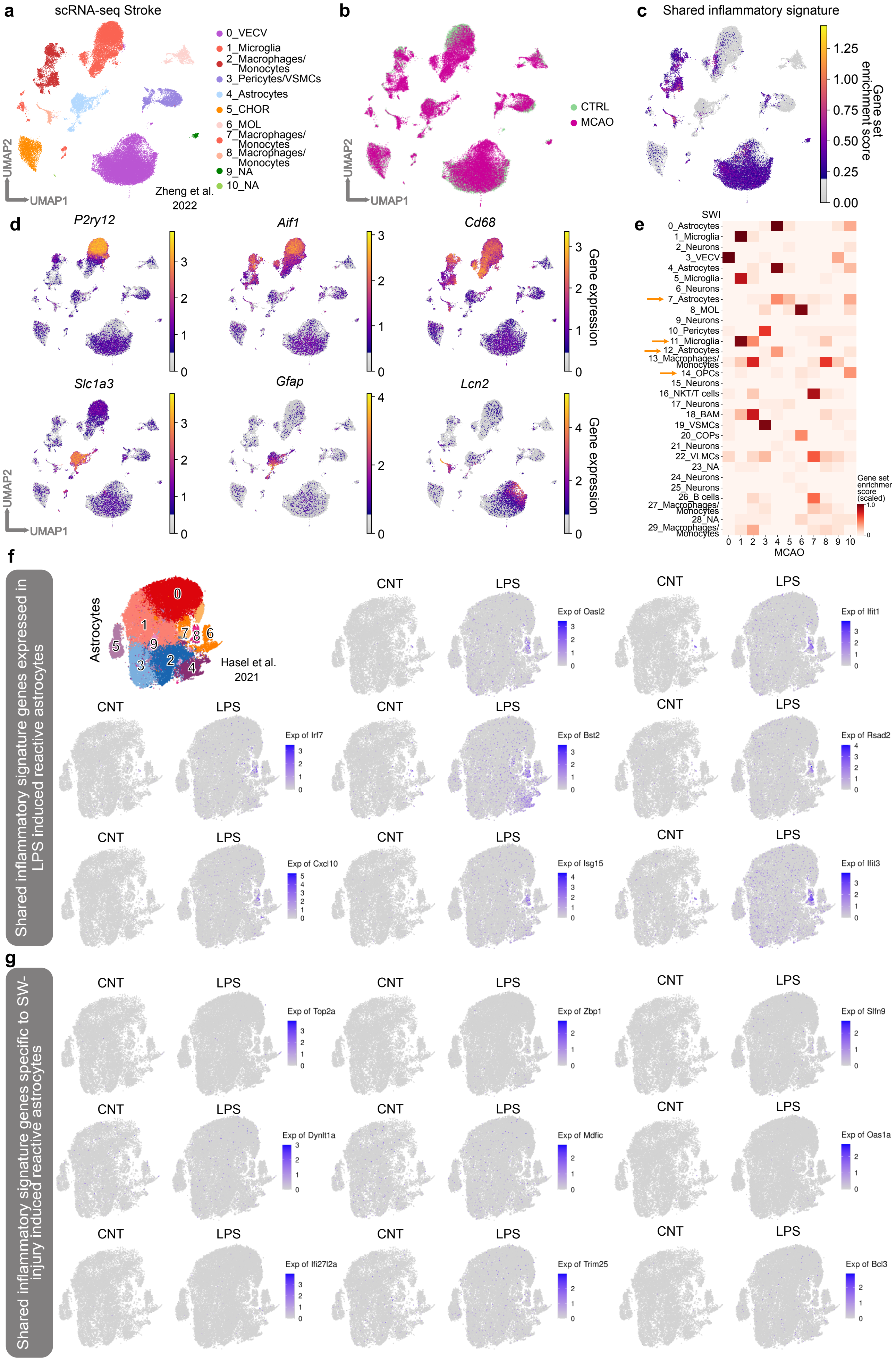
a
scRNA-seq Stroke
0_VECV
1_Microglia
2_Macrophages/Monocytes
3_Pericytes/VSMCs
4_Astrocytes
5_CHOR
6_MOL
7_Macrophages/Monocytes
8_Macrophages/Monocytes
9_NA
10_NA
Zheng et al. 2022
UMAP2
UMAP1
b
CTRL
MCAO
c
Shared inflammatory signature
Gene set enrichment score
d
P2ry12
Aif1
Cd68
Slc1a3
Gfap
Lcn2
Gene expression
e
SWI
0_Astrocytes
1_Microglia
2_Neurons
3_VECV
4_Astrocytes
5_Microglia
6_Neurons
7_Astrocytes
8_MOL
9_Neurons
10_Pericytes
11_Microglia
12_Astrocytes
13_Macrophages/Monocytes
14_OPCs
15_Neurons
16_NKT/T cells
17_Neurons
18_BAM
19_VSMCs
20_COPs
21_Neurons
22_VLMCs
23_NA
24_Neurons
25_Neurons
26_B cells
27_Macrophages/Monocytes
28_NA
29_Macrophages/Monocytes
Gene set enrichmer score (scaled)
MCAO
f
Shared inflammatory signature genes expressed in LPS induced reactive astrocytes
Astrocytes
Hasel et al. 2021
CNT
LPS
Exp of Oasl2
Exp of Ifit1
Exp of Irf7
Exp of Bst2
Exp of Rsad2
Exp of Cxcl10
Exp of Isg15
Exp of Ifit3
g
Shared inflammatory signature genes specific to SW-injury induced reactive astrocytes
Exp of Top2a
Exp of Zbp1
Exp of Slfn9
Exp of Dynlt1a
Exp of Mdfic
Exp of Oas1a
Exp of Ifi27l2a
Exp of Trim25
Exp of Bcl3

Supplementary Figure 9. Common inflammatory gene signature in murine brain cells following MCAO and LPS-induced reactive astrocytes

a-**d** Analysis of MCAO scRNA-seq dataset[51]. **a** UMAP plot illustrating single cells distributed among 11 distinct clusters. **b** UMAP plot depicting the distribution of cells isolated from CTRL and MCAO cerebral cortices. **c** UMAP plot depicting localization of the gene set scores related to the shared inflammatory signature (see Fig. 5c) among all defined cell clusters depicted in Supplementary Fig. 9a. **d** UMAP plots highlighting expression of example marker genes *P2ry12, Aif1, Cd68* to identify reactive microglia clusters and *Slc1a3*, *Gfap* and *Lcn2* to identify reactive astrocyte clusters. **e** Heatmap displaying cluster similarity of SWI dataset (y-axis), and MCAO dataset (x-axis). Orange arrows highlight clusters of interest. **f**-**g** UMAPs illustrating selective marker gene expression of shared inflammatory signature (see Fig. 5c) in subclustered astrocytes based on Hasel et al.[38]. Plots depict presence (**f**) and absence (**g**) of several shared inflammatory genes in reactive astrocyte cluster 8. Abbreviations: UMAP = uniform manifold approximation and projection, MCAO = middle cerebral artery occlusion, CTRL = MCAO sham group, VECV = vascular endothelial cells (venous), CHOR = choroid plexus epithelial cells, MOL = mature oligodendrocytes, OPCs = oligodendrocyte progenitor cells, NKT cells = natural killer T cells, BAM = border-associated macrophages, VSMCs = vascular smooth muscle cells, COPs = committed oligodendrocyte progenitors, VLMCs = vascular and leptomeningeal cells, NA = not available, CNT = control, LPS = lipopolysaccharide.

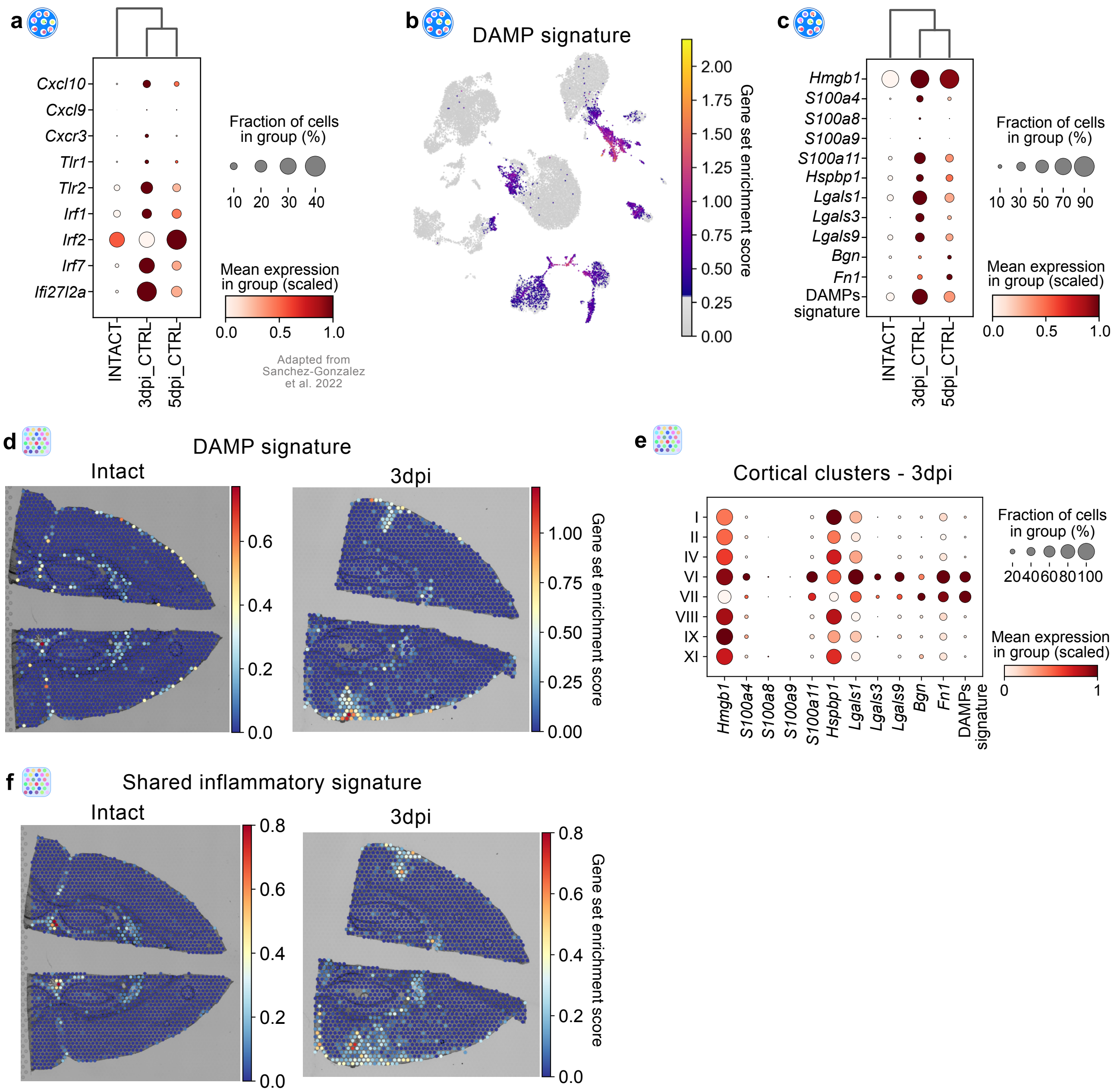

Supplementary Figure 10. Expression of genes associated with Tlr1/2 and Cxcr3 signaling pathways and DAMP signature upon stab wound injury.

a Dot plot depicting gene expression of components associated with the Tlr1/2 and Cxcr3 signaling pathways. Genes are adapted and complemented based on Sanchez-Gonzalez et al.[53]. **b** UMAP plot illustrating the expression of the gene set scores associated with DAMP signature. **c** Dot plot depicting gene expression of components associated with DAMP signature[52] and used to calculate the score in Supplementary Fig. 10b. **d**, **f** Expression of the gene set scores of DAMP (**d**) and shared inflammatory (**f**) signature in spatial context. **e** Dot plot depicting gene expression of components associated with DAMP signature within cortical clusters. Data shown in this figure are derived from n=12 intact animals over 3 independent experiments (5 scRNAseq libraries) , n=3 3dpi animals over 1 experiment (2 scRNAseq libraries), and n=9 5dpi animals over 3 independent experiments (3 scRNAseq libraries). stRNAseq data are based on n=1 animal over 1 experiment with two injured hemispheres being captured. Abbreviations: UMAP = uniform manifold approximation and projection, CTRL = stab wound-injured control animals, DAMPs = Damage-associated molecular patterns.

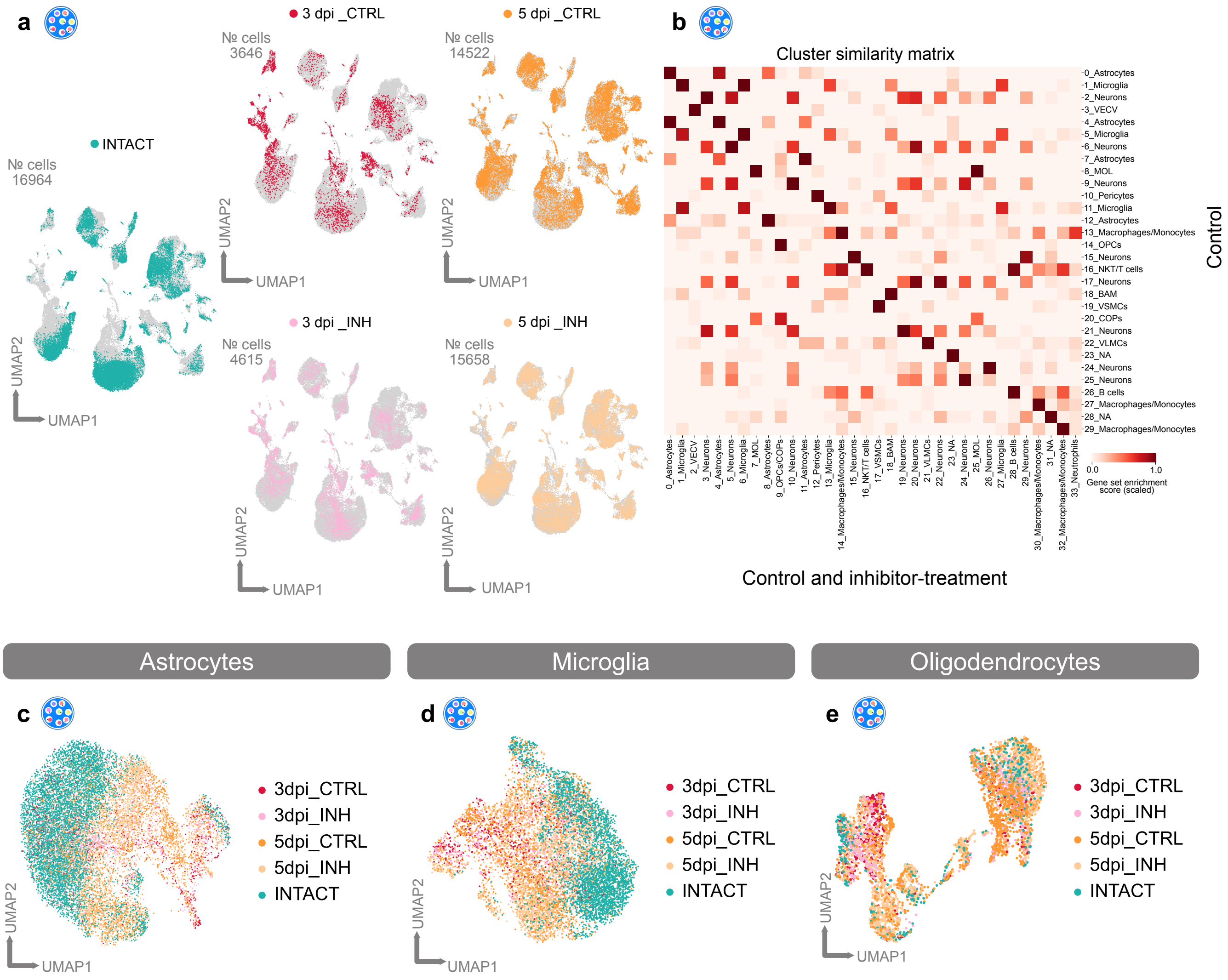

Supplementary Figure 11. scRNA-seq data integration of intact and stab wound-injured cortices.

a UMAP plots depicting cell distribution of integrated and batch-corrected intact (green, 16964 cells), 3 dpi CTRL (red, 3646 cells), 3 dpi INH (pink, 4615 cells), 5 dpi CTRL (orange, 14522 cells), and 5 dpi INH (peach, 15658 cells) datasets. **b** Heatmap displaying cluster similarity between dataset containing integrated control conditions (INT, injured CTRL, y-axis), and dataset containing integrated control and inhibitor treatment conditions (INT, injured CTRL + INH, x-axis). **c** UMAP displaying cell distribution of intact (green), 3 dpi CTRL (red), 3 dpi INH (pink), 5 dpi CTRL (orange), and 5 dpi INH (peach) cells among the 8 identified astrocytic clusters as depicted in Fig. 6c. Clusters AG5, AG6, AG7 and AG8 are mainly formed by cells originating from injured CTRL and INH animals. **d** UMAP displaying cell distribution of intact (green), 3 dpi CTRL (red), 3 dpi INH (pink), 5 dpi CTRL (orange), and 5 dpi INH (peach) cells among the 8 identified microglial clusters as depicted in Fig. 6d. Clusters MG4 and MG6 are mainly formed by cells originating from injured CTRL and INH animals. **e** UMAP displaying cell distribution of intact (green), 3 dpi CTRL (red), 3 dpi INH (pink), 5 dpi CTRL (orange), and 5 dpi INH (peach) cells among the 11 identified oligodendroglial clusters as depicted in Fig. 6e. Cluster OPCs2 is mainly formed by cells originating from injured CTRL and INH animals. Data shown in this figure are derived from n=12 intact animals over 3 independent experiments (5 scRNAseq libraries) , n=3 3dpi CTRL animals over 1 experiment (2 scRNAseq libraries), n=3 3dpi INH animals over 1 experiment (2 scRNAseq libraries), n=9 5dpi CTRL animals over 3 independent experiments (3 scRNAseq libraries) and n=6 5dpi INH animals over 2 independent experiments (3 scRNAseq libraries). Abbreviations: UMAP = uniform manifold approximation and

projection, dpi=days post injury, CTRL = stab wound-injured control mice, INH = stab wound-injured inhibitor-treated mice, VECV = vascular endothelial cells (venous), MOL = mature oligodendrocytes, OPCs = oligodendrocyte progenitor cells, NKT cells = natural killer T cells, BAM = border-associated macrophages, VSMCs = vascular smooth muscle cells, COPs = committed oligodendrocyte progenitors, VLMCs = vascular and leptomeningeal cells, NA = not available.

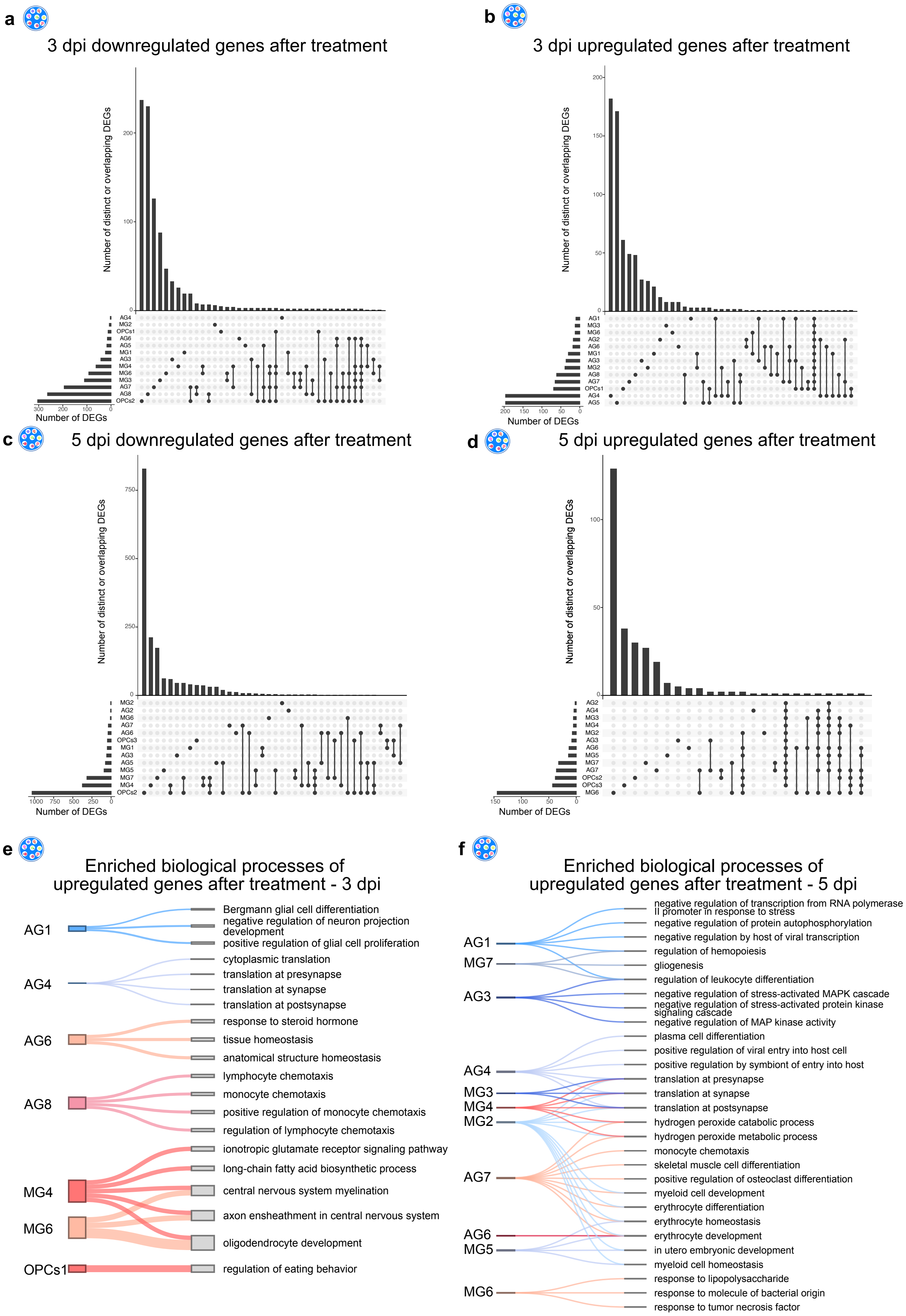
a
3 dpi downregulated genes after treatment
Number of distinct or overlapping DEGs
Number of DEGs
b
3 dpi upregulated genes after treatment
Number of distinct or overlapping DEGs
Number of DEGs
c
5 dpi downregulated genes after treatment
Number of distinct or overlapping DEGs
Number of DEGs
d
5 dpi upregulated genes after treatment
Number of distinct or overlapping DEGs
Number of DEGs
e
Enriched biological processes of upregulated genes after treatment - 3 dpi
AG1
AG4
AG6
AG8
MG4
MG6
OPCs1
Bergmann glial cell differentiation
negative regulation of neuron projection development
positive regulation of glial cell proliferation
cytoplasmic translation
translation at presynapse
translation at synapse
translation at postsynapse
response to steroid hormone
tissue homeostasis
anatomical structure homeostasis
lymphocyte chemotaxis
monocyte chemotaxis
positive regulation of monocyte chemotaxis
regulation of lymphocyte chemotaxis
ionotropic glutamate receptor signaling pathway
long-chain fatty acid biosynthetic process
central nervous system myelination
axon ensheathment in central nervous system
oligodendrocyte development
regulation of eating behavior
f
Enriched biological processes of upregulated genes after treatment - 5 dpi
AG1
MG7
AG3
AG4
MG3
MG4
MG2
AG7
AG6
MG5
MG6
negative regulation of transcription from RNA polymerase II promoter in response to stress
negative regulation of protein autophosphorylation
negative regulation by host of viral transcription
regulation of hemopoiesis
gliogenesis
regulation of leukocyte differentiation
negative regulation of stress-activated MAPK cascade
negative regulation of stress-activated protein kinase signaling cascade
negative regulation of MAP kinase activity
plasma cell differentiation
positive regulation of viral entry into host cell
positive regulation by symbiont of entry into host
translation at presynapse
translation at synapse
translation at postsynapse
hydrogen peroxide catabolic process
hydrogen peroxide metabolic process
monocyte chemotaxis
skeletal muscle cell differentiation
positive regulation of osteoclast differentiation
myeloid cell development
erythrocyte differentiation
erythrocyte homeostasis
erythrocyte development
in utero embryonic development
myeloid cell homeostasis
response to lipopolysaccharide
response to molecule of bacterial origin
response to tumor necrosis factor

Supplementary Figure 12. The Cxcr3 and Tlr1/2 pathway inhibition after stab wound injury induces subcluster specific changes.

a-**d** UpSet plots depicting unique and overlapping downregulated (**a**,**c**) and upregulated (**b**,**d**) DEGs (pval < 0.05, $\log_2$ fold change > 0.7 or $\log_2$ fold change < -0.7, method: Wald test) between different glial subclusters in response to the Cxcr3 and Tlr1/2 inhibition at 3 dpi (**a**,**b**) and 5 dpi (**c**,**d**). Each horizontal bar represents the number of DEGs within a specific cluster, and vertical bars indicate the number of unique or shared genes between clusters. Connected dots highlight shared DEGs across clusters, while single dots represent uniquely regulated DEGs. **e**,**f** Shankey diagram of GO terms linked to glial subclusters illustrating common and unique upregulated biological processes in response to Cxcr3 and Tlr1/2 pathway inhibition at 3 dpi (**e**) and 5 dpi (**f**) (Supplementary Data 7). The top 3 GO terms (based on adjusted p-value) for each glial subcluster are illustrated. The line width indicates the adjusted p-value. Significance was determined by performing GO analysis on gene set. Data shown in this figure are derived from n=12 intact animals over 3 independent experiments (5 scRNAseq libraries) , n=3 3dpi CTRL animals over 1 experiment (2 scRNAseq libraries), n=3 3dpi INH animals over 1 experiment (2 scRNAseq libraries), n=9 5dpi CTRL animals over 3 independent experiments (3 scRNAseq libraries) and n=6 5dpi INH animals over 2 independent experiments (3 scRNAseq libraries). Abbreviations: DEGs = differentially expressed genes, dpi = days post injury, AG = astroglia, MG = microglia, OPCs = oligodendrocyte progenitor cells.

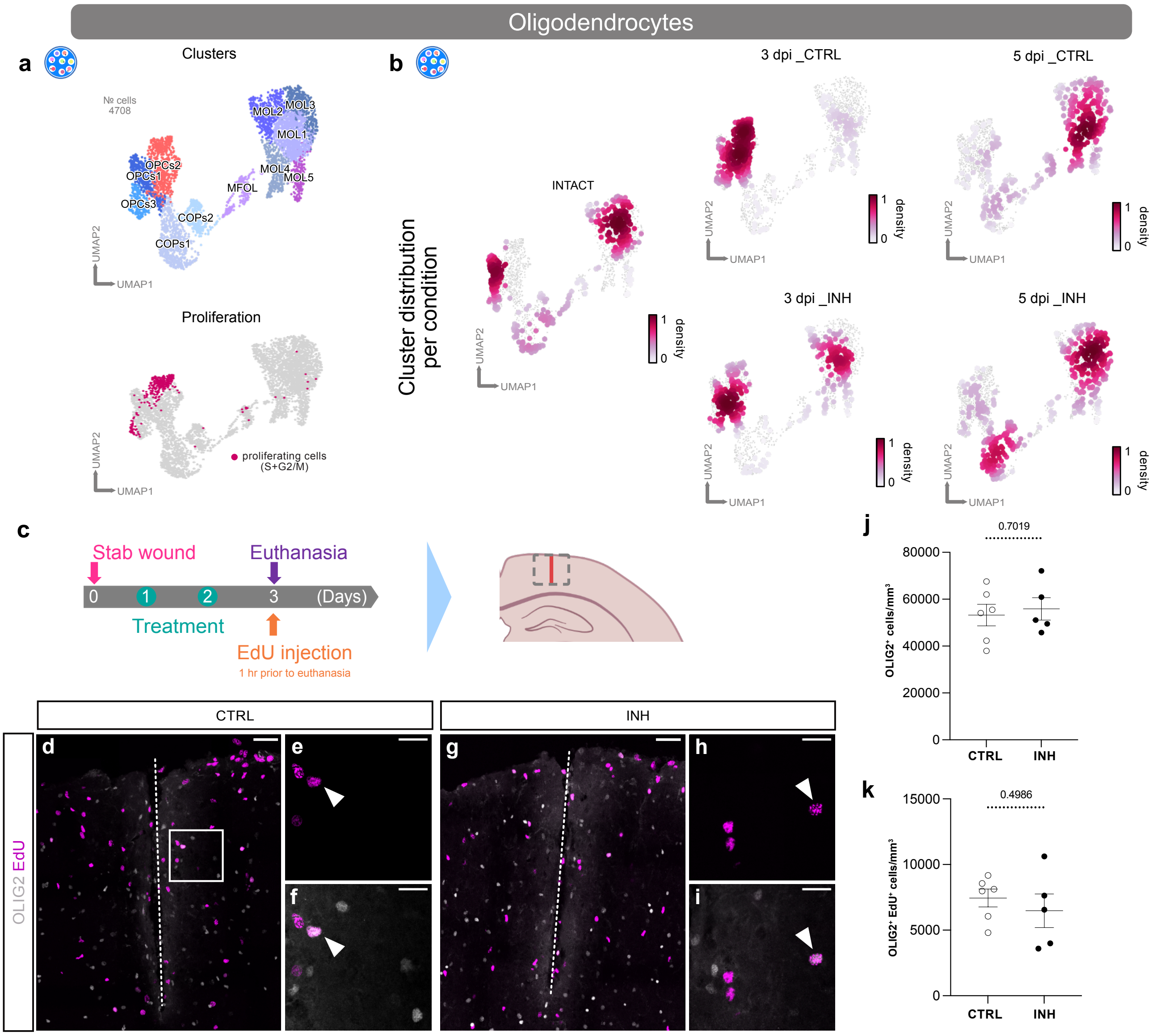

Supplementary Figure 13. OPC reactivity after injury is not altered by the Cxcr3 and Tlr1/2 pathway inhibition.

a,**b** UMAPs illustrating subclusters of oligodendrocytes (**a**) and cell distributions among these subclusters (**b**) at all time points (intact, 3 dpi, and 5 dpi) and conditions (CTRL and INH). Data in (**b**) are downsampled to an equal number of cells between time points and conditions. Data shown in this figure are derived from n=12 intact animals over 3 independent experiments (5 scRNAseq libraries) , n=3 3dpi CTRL animals over 1 experiment (2 scRNAseq libraries), n=3 3dpi INH animals over 1 experiment (2 scRNAseq libraries), n=9 5dpi CTRL animals over 3 independent experiments (3 scRNAseq libraries) and n=6 5dpi INH animals over 2 independent experiments (3 scRNAseq libraries). **c** Experimental paradigm for assessing number of oligodendrocytes and OPC proliferation in injury vicinity. The dashed gray box on mouse brain scheme indicates the analyzed area. The red line highlights the injury core. **d-g** Representative overview images of proliferating OLIG2^{+} (gray) and EdU^{+} (magenta) cells in CTRL (**d**), and INH-treated (**g**) animals. The white dashed lines highlight injury cores. Micrographs in (**e**,**f**,**h**,**i**) are magnifications of the white boxed areas in (**d**) and (**g**), respectively. The white arrowheads in micrographs depict colocalization of EdU (**e**,**h**) with OLIG2^{+} (**f**,**i**) cells. All images are full z-projections of confocal z-stacks. **j**,**k** Dot plots depicting number of oligodendrocytes (OLIG2^{+} cells) (**j**) and proliferating OPCs (OLIG2^{+} and EdU^{+}) (**k**) in CTRL and INH-treated animals. Data are shown as mean ± standard error of the mean. Each data point represents one animal. Source data are provided

as Source Data file. Statistics in (**j**) and (**k**) have been derived from n_{CTRL} = 6 and n_{INH} = 5 animals. p-values were determined with unpaired t-test (two-tailed). Scale bars: **d**,**g**: 50 µm, **e**, **f**, **h**, **i**: 20 µm. Abbreviations: UMAP = uniform manifold approximation and projection, dpi = days post injury, EdU = 5-Ethinyl-2′-deoxyuridine, i.p. = intraperitoneal injection, CTRL = stab wound-injured control animals, INH = stab wound-injured inhibitor-treated animals, OPCs = oligodendrocyte progenitor cells, COPs = committed oligodendrocyte progenitors, MFOL = myelin-forming oligodendrocytes, MOL = mature oligodendrocytes.

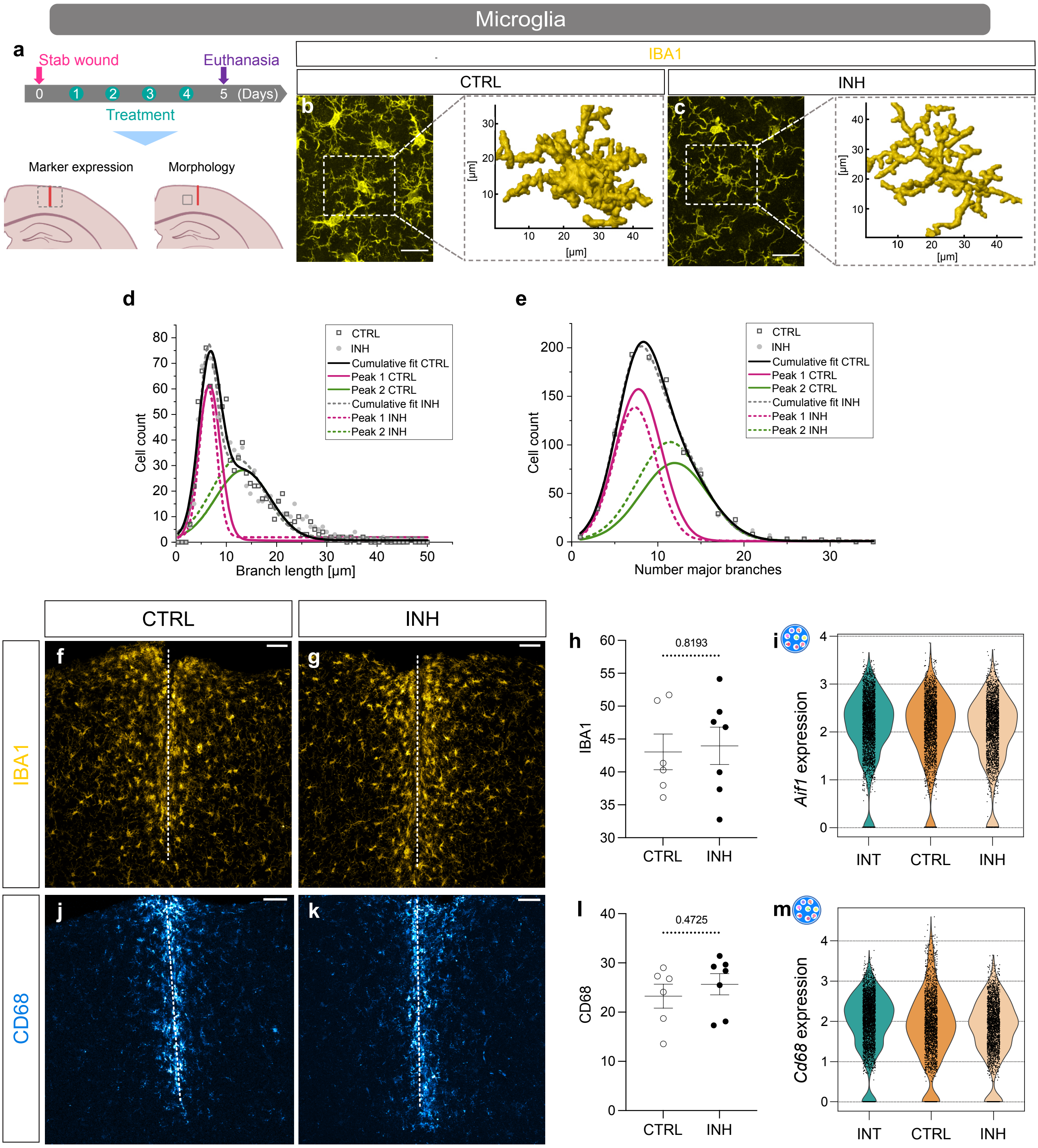

Supplementary Figure 14. Microglia reactivity upon Cxcr3 and Tlr1/2 pathway inhibition.

a Experimental paradigm for assessing microglial reactivity. The gray boxes on the mouse brain scheme highlight the analyzed areas. The red lines indicate the injury cores. **b**,**c** Representative images with 3D reconstruction of IBA1^{+} microglia (yellow) in CTRL (**b**) and INH-treated (**c**) mice. Dashed white boxes indicate selected microglia used for 3D reconstruction. **d**,**e** Plots depict the distribution of microglial branch length (**d**) and number of major branches (**e**) of n=5 CTRL and n=6 INH-treated animals. Source data are provided as Source Data file. Data are fitted with multiple peak functions. The parameters are depicted in Supplementary Data 8 for each fit. **f**,**g**,**j**,**k** Representative

images of IBA1 (**f**,**g**) and CD68 (**j**,**k**) staining in CTRL (**f**,**j**) and INH-treated (**g**,**k**) mice. Dashed white lines indicate the injury cores. All images are full z-projections of confocal z-stacks. **h**,**l** Dot plots depict the mean grey values of $IBA1^{+}$ (**h**) and $CD68^{+}$ (**l**) signal in the injury vicinity of CTRL and INH-treated mice. Data are shown as mean ± standard error of the mean. Each data point represents one animal. Source data are provided as Source Data file. Statistics in (**h**) and (**l**) have been derived from n_{CTLR} = 6 and n_{INH} = 7 animals. p-values were determined with unpaired t-test (two-tailed). **i**,**m** Violin plots depicting expression levels of *Aif1* (**i**) and *Cd68* (**m**) in microglia (scRNA-seq) in intact, 5 dpi CTRL and 5 dpi INH condition. Data shown in violin plot are derived from n=12 intact animals over 3 independent experiments (5 scRNAseq libraries), n=9 5dpi CTRL animals over 3 independent experiments (3 scRNAseq libraries) and n=6 5dpi INH animals over 2 independent experiments (3 scRNAseq libraries). Scale bars: **f**,**g**,**j**,**k**: 50 µm, **b,c**: 20 µm. Abbreviations: dpi = days post injury, CTRL = stab wound- injured control animals, INH = stab wound-injured inhibitor-treated, INT = intact mice.

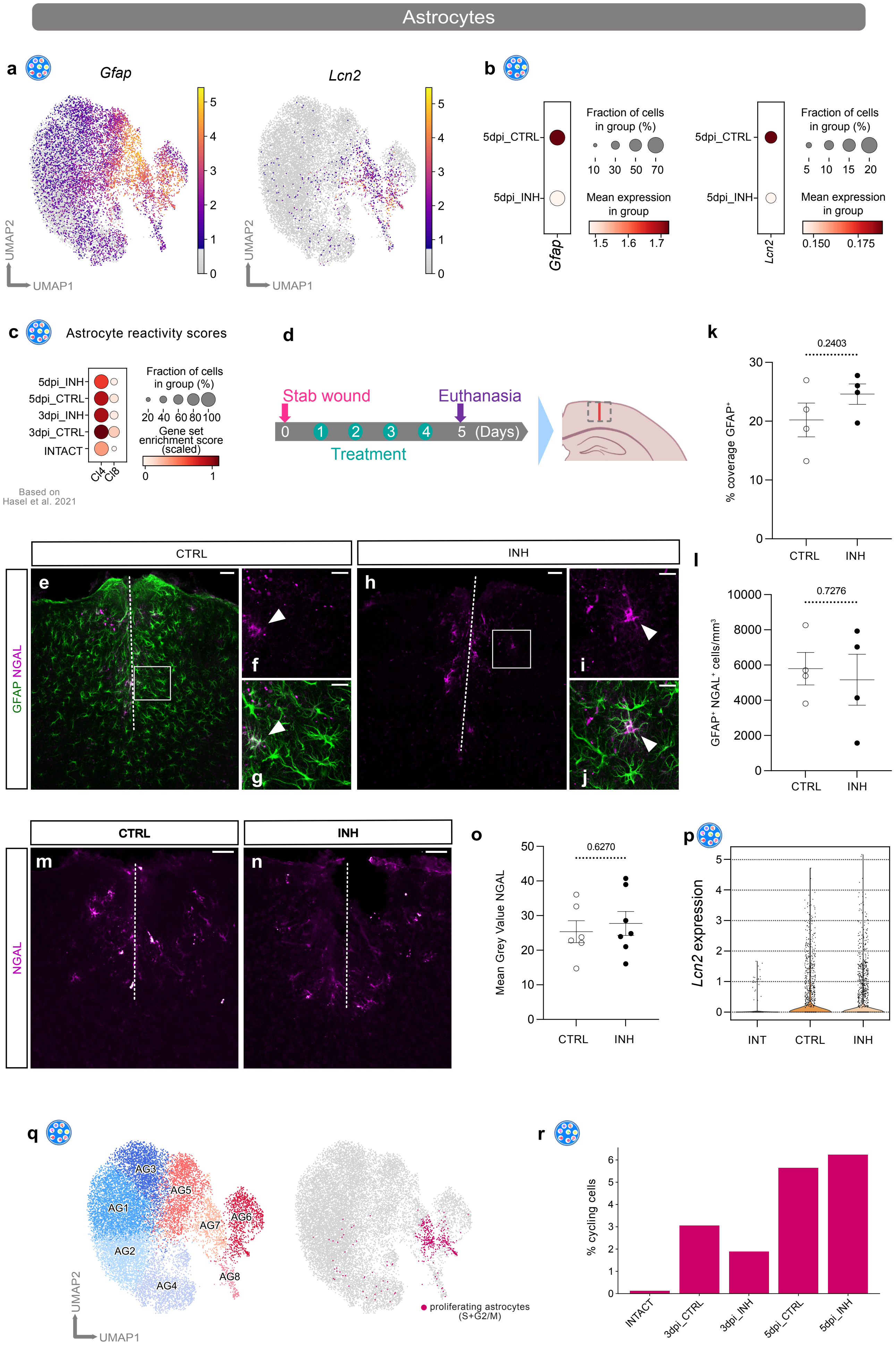
Astrocytes
a
Gfap
Lcn2
UMAP2
UMAP1
0
1
2
3
4
5
b
5dpi_CTRL
5dpi_INH
Gfap
Fraction of cells in group (%)
10 30 50 70
Mean expression in group
1.5 1.6 1.7
Lcn2
5 10 15 20
0.150 0.175
c
Astrocyte reactivity scores
5dpi_INH
5dpi_CTRL
3dpi_INH
3dpi_CTRL
INTACT
Cl4
Cl8
20 40 60 80 100
Gene set enrichment score (scaled)
0
1
Based on Hasel et al. 2021
d
Stab wound
Euthanasia
0 1 2 3 4 5 (Days)
Treatment
k
0.2403
% coverage GFAP+
0
10
20
30
CTRL
INH
CTRL
INH
GFAP NGAL
e
f
g
h
i
j
l
0.7276
GFAP+ NGAL+ cells/mm3
0
2000
4000
6000
8000
10000
CTRL
INH
CTRL
INH
NGAL
m
n
o
0.6270
Mean Grey Value NGAL
0
10
20
30
40
50
CTRL
INH
p
Lcn2 expression
INT
CTRL
INH
q
AG1
AG2
AG3
AG4
AG5
AG6
AG7
AG8
UMAP2
UMAP1
proliferating astrocytes (S+G2/M)
r
% cycling cells
0
1
2
3
4
5
6
INTACT
3dpi_CTRL
3dpi_INH
5dpi_CTRL
5dpi_INH

Supplementary Figure 15. Reaction of astrocytes to stab wound injury in absence of Cxcr3 and Tlr1/2 signaling.

a,**b** UMAP (**a**) and dot plots (**b**) depicting the expression of *Gfap* and *Lcn2* among astrocytic subclusters. **c** Dot plot depicting astrocyte reactivity scores between all time points and conditions. Genes defining astrocyte reactivity scores were extracted from cluster_4 and 8 astrocytes of Hasel et al.[38] (Supplementary Table 7) and are plotted on the reactive subclusters. **d** Experimental paradigm for assessing astrocyte reactivity in the injury vicinity. Dashed gray box on mouse brain scheme indicates analyzed area. Red line highlights the injury core. **e**-**j** Representative images of $GFAP^+$ astrocytes (green) and $NGAL^+$ cells (magenta) in CTRL (**e**) and INH-treated (**h**) animals. White dashed lines highlight injury cores. Micrographs in (**f**,**g**,**i** and **j**) are magnifications of white boxed areas in (**e**) and (**h**). The white arrowheads in micrographs depict colocalization of NGAL with GFAP. All images are full z-projections of confocal z-stacks. **k,l** Dot plots depicting percentage of area covered with $GFAP^+$ signal (**k**) and density of $GFAP^+$ $NGAL^+$ (protein of *Lcn2*) double positive astrocytes (**l**) in CTRL and INH-treated mice. Data are shown as mean ± standard error of the mean. Each data point represents one animal. Source data are provided as Source Data file. Statistics have been derived from n_{CTRL} = 4 and n_{INH} = 4 animals. p-values were determined with unpaired t-test (two-tailed). **m,n** Representative overview images of NGAL distribution (magenta) in CTRL (**m**) and INH-treated (**n**) animals. **o** Dot plot depicting mean grey values of $NGAL^+$ signal of CTRL and INH- treated mice. Data are shown as mean ± standard error of the mean. Each data point represents one animal. Source data are provided as Source Data file. Statistics have been derived from n_{CTRL} = 6 and n_{INH} = 7 animals. p-values were determined with unpaired t-test (two-tailed). **p** Violin plots depicting expression levels of *Lcn2* in astrocytes (scRNA-seq). **q** UMAPs highlighting localization of proliferating astrocytes (pink). **r** Histogram illustrating percentage of proliferating astrocytes (based on S+G2/M score expression (Supplementary Table 4)) between all time points and condition. Data shown in this figure are derived from n=12 intact animals over 3 independent experiments (5 scRNAseq libraries) , n=3 3dpi CTRL animals over 1 experiment (2 scRNAseq libraries), n=3 3dpi INH animals over 1 experiment (2 scRNAseq libraries), n=9 5dpi CTRL animals over 3 independent experiments (3 scRNAseq libraries) and n=6 5dpi INH animals over 2 independent experiments (3 scRNAseq libraries). Scale bars: **e**,**h**,**m**,**n**: 50 µm, **f**, **g**, **i**, **j**: 20 µm. Abbreviations: UMAP = uniform manifold approximation and projection, dpi = days post injury, INT = intact mice, CTRL = stab wound-injured control animals, INH = stab wound-injured inhibitor-treated animals.

Cluster	names	pvals	logFC
I	*Cplx1*	1.89E-124	1.76
	Pvalb	1.85E-106	2.16
	Tmsb10	1.80E-78	1.36
	Mgst3	8.69E-74	1.17
	Serpini1	1.03E-73	1.41
II	*Lamp5*	4.32E-130	2.63
	Calb1	9.14E-134	2.79
	Camk2n1	1.15E-111	1.76
	Rasgrf2	5.02E-117	2.58
	Igfbp6	2.23E-86	2.00
III	*Fth1*	4.75E-311	2.21
	Plp1	4.13E-208	4.56
	Mbp	7.94E-204	3.72
	Mal	3.80E-238	4.44
	Mobp	8.61E-205	3.96
IV	*3110035E14Rik*	*7.17E-97*	*2.24*
	Ttc9b	6.56E-92	2.04
	Rprm	7.36E-93	3.31
	Myl4	4.83E-90	2.85
	Pcp4	7.09E-73	2.31
V	*Cabp7*	2.12E-130	3.63
	Cnih2	7.44E-99	2.21
	Sepw1	2.34E-72	0.91
	Neurod6	6.58E-69	2.54
	Hpca	6.12E-63	2.06
VI	*Serpina3n*	4.79E-72	3.30
	C1qc	4.39E-66	2.65
	Gfap	2.99E-64	2.65
	Tyrobp	6.83E-59	2.38
	Ifi27l2a	1.82E-55	3.78
VII	*Apoe*	1.18E-70	1.30
	mt-Co1	1.41E-58	0.89
	mt-Nd5	1.22E-58	1.17
	mt-Nd2	1.35E-57	1.02
	mt-Co2	4.83E-55	1.02
VIII	*Camk2n1*	4.14E-70	1.90
	Nrsn1	6.58E-66	1.80
	Plcxd2	3.53E-73	2.61
	Rorb	1.36E-66	2.90
	Cnih3	5.63E-65	2.69

Cluster	names	pvals	logFC
IX	*3110035E14Rik*	1.18E-54	2.25
	Rprm	8.12E-45	3.03
	Ttc9b	1.90E-40	1.75
	Pcp4	1.25E-37	2.17
	Cplx3	1.11E-36	4.39
X	*C1ql2*	5.68E-56	5.80
	Fam163b	1.07E-55	3.00
	Ddn	4.44E-49	2.12
	Btbd3	7.11E-51	2.25
	Orai2	8.37E-49	2.81
XI	*Lamp5*	3.16E-24	2.08
	Tshz2	8.22E-23	3.10
	Camk2n2	1.70E-21	1.61
	Nrep	7.83E-22	1.90
	Sparcl1	3.24E-20	0.85
XII	*Calml4*	4.99E-91	7.53
	Ttr	4.93E-111	7.60
	1500015O10Rik	1.15E-92	7.05
	Folr1	4.86E-80	6.67
	Kl	1.91E-70	6.94
XIII	*Spink8*	1.93E-111	5.95
	Tmsb4x	6.09E-68	2.01
	Wipf3	2.23E-64	3.94
	Hpca	4.97E-55	3.62
	Rgs14	1.07E-65	4.47
XIV	*Gpr88*	2.84E-73	5.57
	Ppp1r1b	4.65E-70	4.64
	Pde10a	3.90E-62	5.29
	Penk	1.17E-61	5.64
	Tac1	2.76E-50	5.08
XV	*Calb2*	2.80E-48	6.18
	Ramp3	1.10E-41	4.92
	Tcf7l2	7.64E-35	5.87
	Slc17a6	3.02E-35	4.62
	Zic1	2.69E-32	5.21
XVI	*Tac2*	7.48E-42	8.23
	Gng8	8.03E-34	7.97
	Zic1	6.28E-43	6.42
	Necab2	3.60E-41	5.99
	Gm5741	2.54E-23	9.60

Supplementary Table 1

Top 5 enriched genes in different stRNA-seq identified clusters. Significance was determined using the method t-test overestimated_variance.

Neuronal layers markers[34]	
Layer 2/3	*A830009L08Rik, Gm12371, Lamp5, Calb1, Igfbp6, A830009L08Rik*
Layer 4	*Krt12, Tcap, Dkkl1, Rorb, Tnnc1, A830036E02Rik*
Layer 5	*Hs3st2, Vipr1, Pde1a, Scube1, Ml4, Dkkl1, Fezf2, RP24-134N2.1, Nov, Coro6, Lamp5, Kcng1*
Layer 6	*Rprm, Rell1, Tbr1, 3110035E14Rik, Nxph3, Hs3st4, Myl4, Cpne4, Fezf2, Tshz2, Lypd1*

Supplementary Table 2

List of genes used for each of the cortical neuronal layer gene set scores.

Cell type	Gene	Reference
Microglia	*Tmem119, Cx3cr1, P2ry12, P2ry13, Gpr34, Olfml3, Selplg, Sparc, Fcrls, Siglech, Slc2a5, Mafb, Pmepa1, Cd14*	45, 98, 99,100,101
Disease associated microglia (DAM)	*Lpl, Cst7*	45, 99, 102, 103
Macrophages	*Cd163, Mrc1, Lyve1, Siglec1, Itga4, Tgfbi, Ifitm2, Ifitm3, Tagln2, F13a1, Kynu, S100a11, S100a6*	45,104–106
Border Associated Macrophages (BAM)	*Cd163, Mrc1, Lyve1, Siglec1*	45, 107,108
Monocytes	*Ly6c2, Ccr2, Spn*	109, 110
Dendritic cells (DC)	*Cd24a, Itgax, Bst2, Cd209a, Xcr1, Ccr7, Ccr9, Flt3, Itgae, Itgam, Nudt17, Zbtb46, Ccr9, H2-Oa, Tnni2, Kctd14, Amica1, Kmo, Hepacam2*	45,111, 112, 113, 114
NKT cells	*Cd3e, Klrb1c, Ncr1*	45, 114
T cells	*Cd3e, Lat, Bcl11b*	45, 114
B cells	*Cd19, Ms4a1, Pax5*	45, 114
Neutrophils	*S100a9*	103, 114
Neurons	*Rbfox3, Slc17a7, Gabra1, Cux1, Pou3f2, Bcl11b, Foxp2*	34
OPCs	*Pdgfra, C1ql1*	34
COPs	*Neu4, Brca1*	34
MOL	*Opalin, Ninj2*	34
Astrocytes	*Sox9, Gfap, Aldh1l1, Atp1b2, Vim, Gfap, Slc1a3, Aldoc*	34
Pericytes	*Pecam1, Pdgfrb, Higd1b, Ndufa4l2, Rgs5, Kcnj8, Flt1*	34
VSMCs	*Acta2, Tagln, Myh11, Mustn1, Map3k7cl*	34
VLMCs	*Mgp, Slc47a1, Thbd, Col1a1, Col1a2*	34
VECV	*Ly6c1, Cldn5, Ly6a, Pltp, Pglyrp1, Scgb3a1*	34

Supplementary Table 3

List of marker genes of well-characterized cell populations, employed for cluster identification.

Cell Cycle genes[46, 47]			
S-phase		G2M-phase	
Mcm5	*Atad2*	*Hmgb2*	*Hjurp*
Pcna	*Rad51*	*Cdk1*	*Cdca3*
Tyms	*Rrm2*	*Nusap1*	*Hn1*
Fen1	*Cdc45*	*Ube2c*	*Cdc20*
Mcm2	*Cdc6*	*Birc5*	*Ttk*
Mcm4	*Exo1*	*Tpx2*	*Cdc25c*
Rrm1	*Tipin*	*Top2a*	*Kif2c*
Ung	*Dscc1*	*Ndc80*	*Rangap1*
Gins2	*Blm*	*Cks2*	*Ncapd2*
Mcm6	*Casp8ap2*	*Nuf2*	*Dlgap5*
Cdca7	*Usp1*	*Cks1b*	*Cdca2*
Dtl	*Clspn*	*Mki67*	*Cdca8*
Prim1	*Pola1*	*Tmpo*	*Ect2*
Uhrf1	*Chaf1b*	*Cenpf*	*Kif23*
Mlf1ip	*Brip1*	*Tacc3*	*Hmmr*
Hells	*E2f8*	*Fam64a*	*Aurka*
Rfc2		*Smc4*	*Psrc1*
Rpa2		*Ccnb2*	*Anln*
Nasp		*Ckap2l*	*Lbr*
Rad51ap1		*Ckap2*	*Ckap5*
Gmnn		*Aurkb*	*Cenpe*
Wdr76		*Bub1*	*Ctcf*
Slbp		*Kif11*	*Nek2*
Ccne2		*Anp32e*	*G2e3*
Ubr7		*Tubb4b*	*Gas2l3*
Pold3		*Gtse1*	*Cbx5*
Msh2		*Kif20b*	*Cenpa*

Supplementary Table 4

List of genes used to calculate the cell cycle phase score.

	Shared inflammatory genes	
	3dpi - downregulated	5dpi- downregulated
AG1	-	-
AG2	-	*Ifitm3*
AG3	-	-
AG4	-	-
AG5	*Rsad2, Ifit3, Ifit3b, Bst2*	*Ifitm3, Bst2*
AG6	*Isg15, Bst2, Rtp4, Ifit3, Ifit3b*	Isg15
AG7	*Isg15, Cxcl10, Oasl2, Ifitm3, Irf7, Bst2, Trim25, Rsad2, Rtp4, Ifit3, Ifit3b, Ifit1*	*Isg15, Irf7, Ifi27l2a, Rtp4*
AG8	*Rsad2, Ifi27l2a*	-
MG1	*Ifitm3*	-
MG2	-	*Ifitm3*
MG3	*Isg15, Ifitm3, Bst2, Ifi27l2a, Rtp4, Ifit3*	
MG4	*Zbp1, Isg15, Cxcl10, Oasl2, Oasl1, Oas1a, Ifitm3, Irf7, Bst2, Rsad2, Rtp4, Ifit3, Ifit3b*	*Isg15, Cxcl10, Ifitm2, Irf7, Bst2, Rsad2, Lgals1, Ifit3, Ifit1*
MG5	-	*Isg15, Ifitm3, Irf7*
MG6	*Zbp1, Isg15, Oasl2, Oas1a, Ifitm3, Irf7, Bst2, Dhx58, Ifi27l2a, Rtp4, Ifit3*	-
MG7	-	*Bst2, Ifi27l2a, Lgals1*
MG8	-	-
OPCs1	*Ifitm3*	-
OPCs2	*Isg15, Oasl2, Oas1a, Ifitm3, Irf7, Bst2, Rsad2, Ifi27l2a, Lgals1, Rtp4, Ifit3, Ifit3b, Ifit1*	*Bcl3, Ifitm2, Ifitm3, Bst2, Ifi27l2a, Lgals1*
OPCs3	-	-

Supplementary Table 5

List of shared inflammatory genes which are downregulated after INH treatment in each subcluster.

Sample-id (Matrix)	FastQ	Condition	Chromium Kit	Sequencing plattform	cellranger	number of cells	mean genes	mean counts
21L008532	21L008532, 22L000025	5dpi_CTRL	V3.1	Novaseq6000	6.0.0	7440	3212	10960
21L008533	21L008533, 22L000026	5dpi_INH	V3.1	Novaseq6000	6.0.0	7403	3334	11696
MUC29190	MUC29190	Intact	V3.1	Novaseq6000	6.0.0	4357	2595	7301
MUC18415	MUC18415	5dpi_CTRL	V2	Novaseq6000	3.0.2	4615	2759	9150
MUC13721	MUC13721	Intact	V2	HiSeq 3000/4000	3.0.2	2674	2121	5674
MUC13722	MUC13722	3dpi_CTRL	V2	HiSeq 3000/4000	3.0.2	1848	2306	7934
MUC13723	MUC13723	3dpi_CTRL	V2	HiSeq 3000/4000	3.0.2	1795	2403	8731
MUC13724	MUC13724	3dpi_INH	V2	HiSeq 3000/4000	3.0.2	1865	2377	8036
MUC13725	MUC13725	3dpi_INH	V2	HiSeq 3000/4000	3.0.2	2748	2420	8259
MUC13726	MUC13726	Intact	V2	HiSeq 3000/4000	3.0.2	2471	2069	5360
MUC13727	MUC13727	5dpi_CTRL	V2	HiSeq 3000/4000	3.0.2	1711	2385	7665
MUC13729	MUC13729	5dpi_INH	V2	HiSeq 3000/4000	3.0.2	3831	2199	6306
MUC13730	MUC13730	5dpi_INH	V2	HiSeq 3000/4000	3.0.2	3908	2125	6063
MUC13731	MUC13731	Intact	V2	HiSeq 3000/4000	3.0.2	3385	1764	4443
MUC13732	MUC13732	Intact	V2	HiSeq 3000/4000	3.0.2	3762	1782	4505

Supplementary Table 6

Specification of datasets used for the scRNA-seq analysis.

Genes defining astrocyte reactivity scores - extracted from cluster_4 and 8 astrocytes of Hasel et al.[38]	
Cl4	Cl8
Lcn2	*Igtp*
Ifitm3	*Gm4951*
H2-D1	*Iigp1*
Timp1	*Irgm1*
B2m	*Tap1*
Vim	*Ifit3*
Gfap	*Gbp2*
Psmb8	*F830016B08Rik*
Bst2	*Gbp7*
H2-T23	*Tap2*
Cd63	*Ifi47*
Serping1	*B2m*
Tspo	*Ifit1*
Rps4x	*Psmb8*
S100a6	*Stat1*
Tuba1a	*H2-T23*
H2-K1	*Usp18*
Mt1	*Ifit3b*
Gpx1	*Rtp4*
S100a11	*Psmb9*

Supplementary Table 7

List of genes used to calculate the gene set score in Supplementary Fig. 15c.

References:

34. Zeisel, A. *et al.* Molecular Architecture of the Mouse Nervous System. *Cell* **174**, 999-1014. e22 (2018).
38. Hasel, P., Rose, I. V. L., Sadick, J. S., Kim, R. D. & Liddelow, S. A. Neuroinflammatory astrocyte subtypes in the mouse brain. *Nat Neurosci* **24**, 1475–1487 (2021).
45. Ochocka, N. *et al.* Single-cell RNA sequencing reveals functional heterogeneity of glioma-associated brain macrophages. *Nat Commun* **12**, 1151 (2021).
47. Tirosh, I. *et al.* Dissecting the multicellular ecosystem of metastatic melanoma by single-cell RNA-seq. *Science (1979)* **352**, 189–196 (2016).
46. Baranek, T. *et al.* High Dimensional Single-Cell Analysis Reveals iNKT Cell Developmental Trajectories and Effector Fate Decision. *Cell Rep* **32**, 108116 (2020).
51. Zheng, K. *et al.* Single-cell RNA-seq reveals the transcriptional landscape in ischemic stroke. *Journal of Cerebral Blood Flow and Metabolism* **42**, (2022).
52. Midwood, K. S. & Piccinini, A. M. DAMPening inflammation by modulating TLR signalling. *Mediators Inflamm* 2010, (2010).
53. Sanchez-Gonzalez, R. *et al.* Innate Immune Pathways Promote Oligodendrocyte Progenitor Cell Recruitment to the Injury Site in Adult Zebrafish Brain. *Cells* **11**, 520 (2022).
98. Sousa, C., Biber, K. & Michelucci, A. Cellular and Molecular Characterization of Microglia: A Unique Immune Cell Population. *Front Immunol* **8**, (2017).
99. Masuda, T. *et al.* Spatial and temporal heterogeneity of mouse and human microglia at single-cell resolution. *Nature* **566**, 388–392 (2019).
100. Bennett, M. L. *et al.* New tools for studying microglia in the mouse and human CNS. *Proceedings of the National Academy of Sciences* **113**, E1738–E1746 (2016).
101. Matcovitch-Natan, O. *et al.* Microglia development follows a stepwise program to regulate brain homeostasis. *Science (1979)* **353**, aad8670 (2016).
102. Keren-Shaul, H. *et al.* A Unique Microglia Type Associated with Restricting Development of Alzheimer's Disease. *Cell* **169**, 1276-1290.e17 (2017).
103. Li, Y. *et al.* Microglia-organized scar-free spinal cord repair in neonatal mice. *Nature* (2020) doi:10.1038/s41586-020-2795-6.
104. Venteicher, A. S. *et al.* Decoupling genetics, lineages, and microenvironment in IDH-mutant gliomas by single-cell RNA-seq. *Science (1979)* **355**, eaai8478 (2017).
105. Müller, S. *et al.* Single-cell profiling of human gliomas reveals macrophage ontogeny as a basis for regional differences in macrophage activation in the tumor microenvironment. *Genome Biol* **18**, 234 (2017).
106. Bowman, R. L. *et al.* Macrophage Ontogeny Underlies Differences in Tumor-Specific Education in Brain Malignancies. *Cell Rep* **17**, 2445–2459 (2016).
107. Jordão, M. J. C. *et al.* Single-cell profiling identifies myeloid cell subsets with distinct fates during neuroinflammation. *Science (1979)* **363**, eaat7554 (2019).
108. Goldmann, T. *et al.* Origin, fate and dynamics of macrophages at central nervous system interfaces. *Nat Immunol* **17**, 797–805 (2016).
109. Ziegler-Heitbrock, L. *et al.* Nomenclature of monocytes and dendritic cells in blood. *Blood* **116**, e74–e80 (2010).
110. Thomas, G. D. *et al.* Human Blood Monocyte Subsets. *Arterioscler Thromb Vasc Biol* **37**, 1548–1558 (2017).
111. Fleming, T. J., Fleming, M. L. & Malek, T. R. Selective expression of Ly-6G on myeloid lineage cells in mouse bone marrow. RB6-8C5 mAb to granulocyte-differentiation antigen (Gr-1) detects members of the Ly-6 family. *The Journal of Immunology* **151**, 2399 (1993).
112. Musumeci, A., Lutz, K., Winheim, E. & Krug, A. B. What makes a PDC: Recent advances in understanding plasmacytoid DC development and heterogeneity. *Frontiers in Immunology*. vol. 10 Preprint at https://doi.org/10.3389/fimmu.2019.01222 (2019).
113. Hey, Y. & O'Neill, H. C. Antigen Presenting Properties of a Myeloid Dendritic-Like Cell in Murine Spleen. *PLoS One* **11**, e0162358- (2016).
114. Van Hove, H. *et al.* A single-cell atlas of mouse brain macrophages reveals unique transcriptional identities shaped by ontogeny and tissue environment. *Nat Neurosci* **22**, 1021–1035 (2019).
