# Supplementary material for: Shared inflammatory glial cell signature after stab wound injury, revealed by spatial, temporal, and cell-type-specific profiling of the murine cerebral cortex
Source: Nat Commun. 2024 Apr 3;15:2866. doi: 10.1038/s41467-024-46625-w (PMC10991294; doi:10.1038/s41467-024-46625-w)
Supplement: Supplementary file 3 — Description of Additional Supplementary Files [file 41467_2024_46625_MOESM3_ESM.pdf]

Description of Additional Supplementary Files

Supplementary Data 1
Cluster VI significantly enriched genes (pval < 0.05, log2 fold change > 1) and GO term over-represented in cluster VI. Significance was determined using the GO enrichment analysis on gene sets.

Supplementary Data 2
Gene set enrichment analysis along the spatial gradient. Significance was determined using GSEA by hypergeometric testing within the groups.

Supplementary Data 3
Top 10 enrichment genes across the 30 scRNA-seq clusters of the UMAP in Fig.3b. Significance was determined using the "t-test overestimated_variance" method.

Supplementary Data 4
Top 20 enrichment genes across the 30 scRNA-seq clusters of the UMAP in Fig.4b. Significance was determined using the "t-test overestimated_variance" method.

Supplementary Data 5
Differentially expressed genes (DEGs) of each glial subcluster (pval < 0.05, log2 fold change > 1.6 or < -1.6). DEGs are determined by comparing each glial subcluster to all other subclusters within the respective cell type. Significance was determined using the "t-test overestimated_variance." method.

Supplementary Data 6
GO term analysis of the 241 commonly upregulated genes from the comparison of clusters MG4, AG5, and OPCs2. Significance was determined using the GO enrichment analysis on gene sets.

Supplementary Data 7
GO term analysis of the DEGs between INH and CTRL conditions in each cluster and at each timepoint. Significance was determined by performing GO enrichment analysis on gene sets.

Supplementary Data 8
List of the fit parameters for the distribution of microglia cells, obtained by applying the multipack function to the extracted parameters (Fig. 7d-g, Supplementary Fig 14d,e). The data distributions are compared using Compare Datasets: fitcmpdata function in Origin.
