# Supplementary material for: Shared inflammatory glial cell signature after stab wound injury, revealed by spatial, temporal, and cell-type-specific profiling of the murine cerebral cortex
Source: Nat Commun. 2024 Apr 3;15:2866. doi: 10.1038/s41467-024-46625-w (PMC10991294; doi:10.1038/s41467-024-46625-w)
Supplement: Supplementary file 11 — Supplementary Data 8 [file 41467_2024_46625_MOESM11_ESM.pdf]

Supplementary Data 8

Statistics for comparison of circularity (related to Fig. 7d)

Fit Comparison of Data				
Description				
Input Data	CTRL<br>Source Data<br>TAB: Fig. 7d-g<br>Column: circularity		INH<br>Source Data<br>TAB: Fig. 7d-g<br>Column: circularity	
Fit Report	Fit Report CTRL (see bellow)		Fit Report INH (see bellow)	
Equation	$y = y_0 + A * e^{-\frac{1}{2}*(\frac{x-x_c}{w})^2}$		$y = y_0 + A * e^{-\frac{1}{2}*(\frac{x-x_c}{w})^2}$	
Function	GaussAmp		GaussAmp	
Number of Points	68		68	
Number of Parameters	10		10	
F-test				
	F	Numer.DF	Denom.DF	Prob > F
	7.269	10	116	$7.630e^{-9}$
At the 0.05 significance level, the two datasets are significantly different.				

Fit Reports			CTRL					INH				
	Peak	Parameter	Value	Standard Error	t-Value	Prob>|t|	Dependency	Value	Standard Error	t-Value	Prob>|t|	Dependency
Fit Parameters	Peak 1	y_0	0.181	0.596	0.303	0.762	0.524	0.938	0.565	1.659	0.102	0.421
		x_c	0.037	0.001	24.45	$3e^{-32}$	0.928	0.032	$5.6e^{-4}$	57.42	$8e^{-53}$	0.777
		w	0.012	$9.5e^{-4}$	12.63	$2e^{-18}$	0.879	0.007	$4.9e^{-4}$	15.58	$2e^{-22}$	0.802
		A	69.06	9.363	7.376	$6e^{-10}$	0.969	86.91	5.802	14.97	$1e^{-21}$	0.862
		FWHM	0.028	0.002	-	-	-	0.018	0.001	-	-	-
		Area	2.079	0.419	-	-	-	1.674	0.173	-	-	-
	Peak 2	y_0	0.181	0.596	0.303	0.762	0.524	0.938	0.565	1.659	0.102	0.421
		x_c	0.071	0.005	13.93	$3e^{-20}$	0.968	0.055	0.001	31.31	$4e^{-38}$	0.762
		w	0.017	0.007	2.285	0.099	0.991	0.008	0.002	3.439	0.001	0.923
		A	37.59	21.62	1.738	0.087	0.996	28.86	11.04	2.613	0.011	0.967
		FWHM	0.042	0.018	-	-	-	0.020	0.006	-	-	-
		Area	1.683	1.659	-	-	-	0.638	0.392	-	-	-
	Peak 3	y_0	0.181	0.596	0.303	0.762	0.524	0.938	0.565	1.659	0.12	0.421
		x_c	0.110	0.044	2.461	0.016	0.994	0.079	0.007	10.66	$2e^{-15}$	0.971
		w	0.025	0.020	1.203	0.233	0.984	0.023	0.004	5.145	$3e^{-6}$	0.952
		A	12.60	12.02	1.048	0.298	0.991	33.01	3.642	9.062	$1e^{-12}$	0.888
		FWHM	0.059	0.049	-	-	-	0.056	0.010	-	-	-
		Area	0.791	1.380	-	-	-	1.983	0.552	-	-	-

		CTRL	INH
Fit Statistics	Number of Points	68	68
	Degrees of Freedom	58	58
	Reduced Chi-Sqr	11.51	12.56
	Residual Sum of Squares	667.5	728.9
	R-Square (COD)	0.977	0.978
	Adj. R-Square	0.974	0.974

Statistics for comparison of soma volume (related to Fig. 7e)

Fit Comparison of Data		
**Description**		
Input Data	CTRL<br>Source Data<br>TAB: Fig. 7d-g<br>Column: somaVol	INH<br>Source Data<br>TAB: Fig. 7d-g<br>Column: somaVol
Fit Report	Fit Report CTRL (see bellow)	Fit Report INH (see bellow)
Equation	$y = y_0 + A * e^{-\frac{1}{2}*(\frac{x-x_c}{w})^2}$	$y = y_0 + A * e^{-\frac{1}{2}*(\frac{x-x_c}{w})^2}$
Function	GaussAmp	GaussAmp
Number of Points	250	250
Number of Parameters	7	7

F-test				
	F	Numer.DF	Denom.DF	Prob > F
	13.44	7	486	$6.66e^{-16}$
At the 0.05 significance level, the two datasets are significantly different.				

Fit Reports			CTRL					INH				
	Peak	Parameter	Value	Standard Error	t-Value	Prob>|t|	Dependency	Value	Standard Error	t-Value	Prob>|t|	Dependency
Fit Parameters	Peak 1	y_0	0.299	0.125	2.380	0.018	0.162	0.206	0.149	1.381	0.168	0.201
		x_c	221.2	1.791	123.5	$2e^{-221}$	0.274	210.1	2.544	82.60	$7e^{-180}$	0.374
		w	53.66	2.277	23.56	$1e^{-64}$	0.700	83.93	5.386	15.58	$1.9e^{-38}$	0.905
		A	41.52	1.455	28.52	$1e^{-79}$	0.670	45.85	3.691	12.42	$9.3e^{-28}$	0.955
		FWHM	126.3	5.362	-	-	-	197.6	12.68	-	-	-
		Area	5586	343.1	-	-	-	9647	1335	-	-	-
	Peak 2	y_0	0.299	0.125	2.380	0.018	0.162	0.206	0.149	1.381	0.168	0.201
		x_c	327.8	7.961	41.18	$1e^{-111}$	0.655	422.0	48.85	8.639	$7.6e^{-16}$	0.959
		w	183.4	5.787	31.69	$2e^{-88}$	0.521	212.0	27.32	7.763	$2.3e^{-13}$	0.908
		A	25.68	1.083	23.71	$3e^{-65}$	0.825	15.05	1.475	10.20	$1.4e^{-20}$	0.890
		FWHM	431.9	13.62	-	-	-	499.4	64.33	-	-	-
		Area	11811	477.5	-	-	-	8002	1635	-	-	-

		CTRL	INH
Fit Statistics	Number of Points	250	250
	Degrees of Freedom	243	243
	Reduced Chi-Sqr	3.31595	4.47978
	Residual Sum of Squares	805.77531	1088.58534
	R-Square (COD)	0.97181	0.95924
	Adj. R-Square	0.97112	0.95823

Statistics for comparison of Branch volume (related to Fig. 7f)

Fit Comparison of Data				
Description				
Input Data	CTRL<br>Source Data<br>TAB: Fig. 7d-g<br>Column: branchVol		INH<br>Source Data<br>TAB: Fig. 7d-g<br>Column: branchVol	
Fit Report	Fit Report CTRL (see bellow)		Fit Report INH (see bellow)	
Equation	$y = y_0 + A * e^{-\frac{1}{2}*(\frac{x-x_c}{w})^2}$		$y = y_0 + A * e^{-\frac{1}{2}*(\frac{x-x_c}{w})^2}$	
Function	GaussAmp		GaussAmp	
Number of Points	150		150	
Number of Parameters	7		7	
F-test				
	F	Numer.DF	Denom.DF	Prob > F
	9.239	7	201	$6.695e^{-10}$
At the 0.05 significance level, the two datasets are significantly different.				

Fit Reports												
			CTRL					INH				
	Peak	Parameter	Value	Standard Error	t-Value	Prob>|t|	Dependency	Value	Standard Error	t-Value	Prob>|t|	Dependency
Fit Parameters	Peak 1	y_0	0.521	0.203	2.563	0.011	0.144	0.179	0.515	0.347	0.729	0.447
		x_c	651.2	11.95	54.46	$1e^{-97}$	0.836	758.0	20.16	37.59	$2e^{-42}$	0.913
		w	221.5	22.79	9.718	$1e^{-17}$	0.969	316.9	24.30	13.03	$6e^{-19}$	0.959
		A	75.31	20.18	3.731	$2.7e^{-4}$	0.996	98.67	20.83	4.735	$1e^{-5}$	0.996
		FWHM	521.6	53.67	-	-	-	746.3	57.24	-	-	-
		Area	41818	15362	-	-	-	78387	22267	-	-	-
	Peak 2	y_0	0.521	0.203	2.563	0.011	0.144	0.179	0.515	0.347	0.729	0.447
		x_c	1058	142.4	7.431	$8e^{-12}$	0.994	1403.5	957.8	1.465	0.148	0.994
		w	372.0	59.69	6.232	$4.8e^{-9}$	0.980	520.3	464.9	1.119	0.267	0.983
		A	45.90	10.08	4.550	$1.1e^{-5}$	0.992	10.22	9.086	1.125	0.265	0.987
		FWHM	876.1	140.5	-	-	-	1225	1094	-	-	-
		Area	42814	15919	-	-	-	13333	23254	-	-	-
Fit Statistics	Number of Points		150					65				
	Degrees of Freedom		143					58				
	Reduced Chi-Sqr		5.31038					9.53496				
	Residual Sum of Squares		759.38391					553.02786				
	R-Square (COD)		0.98577					0.98921				
	Adj. R-Square		0.98518					0.98809				

Statistics for comparison of Number of Nodes per major branch (related to Fig. 7fg

Fit Comparison of Data				
Description				
Input Data	CTRL<br>Source Data<br>TAB: Fig. 7d-g<br>Column: branchNodesPerBranch		INH<br>Source Data<br>TAB: Fig. 7d-g<br>Column: branchNodesPerBranch	
Fit Report	Fit Report CTRL (see bellow)		Fit Report INH (see bellow)	
Equation	$y = y_0 + A * e^{-\frac{1}{2}*(\frac{x-x_c}{w})^2}$		$y = y_0 + A * e^{-\frac{1}{2}*(\frac{x-x_c}{w})^2}$	
Function	GaussAmp		GaussAmp	
Number of Points	60		50	
Number of Parameters	7		7	
F-test				
	F	Numer.DF	Denom.DF	Prob > F
	4.120	7	96	$5.343e^{-4}$
At the 0.05 significance level, the two datasets are significantly different.				

Fit Reports			CTRL					INH				
	Peak	Parameter	Value	Standard Error	t-Value	Prob>|t|	Dependency	Value	Standard Error	t-Value	Prob>|t|	Dependency
Fit Parameters	Peak 1	y_0	1.314	0.802	1.638	0.107	0.294	2.432	1.022	2.380	0.021	0.338
		x_c	7.810	0.162	47.95	$2e^{-45}$	0.306	7.555	0.111	67.48	$2e^{-45}$	0.139
		w	2.822	0.314	8.988	$3e^{-12}$	0.875	2.059	0.172	11.93	$3e^{-15}$	0.758
		A	97.151	13.76	7.055	$3.6e^{-9}$	0.942	122.1	8.966	13.61	$3e^{-17}$	0.764
		FWHM	6.646	0.739	-	-	-	4.850	0.406	-	-	-
		Area	687.3	163.8	-	-	-	630.1	83.72	-	-	-
	Peak 2	y_0	1.314	0.802	1.638	0.107	0.294	2.432	1.022	2.380	0.021	0.338
		x_c	16.39	1.476	11.10	$1e^{-15}$	0.945	15.18	0.950	15.96	$1e^{-19}$	0.817
		w	6.790	1.016	6.681	$1.4e^{-8}$	0.921	6.862	0.716	9.573	$3e^{-12}$	0.778
		A	59.35	4.140	14.33	$7e^{-20}$	0.735	57.18	3.637	15.72	$1e^{-19}$	0.569
		FWHM	15.98	2.392	-	-	-	16.15	1.687	-	-	-
		Area	1010	194.1	-	-	-	983.5	123.2	-	-	-
Fit Statistics		Number of Points	60					50				
		Degrees of Freedom	53					43				
		Reduced Chi-Sqr	27.25					34.58				
		Residual Sum of Squares	1444					1486				
		R-Square (COD)	0.972					0.973				
		Adj. R-Square	0.969					0.969				

Statistics for comparison of Branch length (related to Suppl. Fig. 14d)

Fit Comparison of Data				
Description				
Input Data	CTRL<br>Source Data<br>TAB: Fig. 7d-g<br>Column: branchLengthSkel_3		INH<br>Source Data<br>TAB: Fig. 7d-g<br>Column: branchLengthSkel_3	
Fit Report	Fit Report CTRL (see bellow)		Fit Report INH (see bellow)	
Equation	$y = y_0 + A * e^{-\frac{1}{2}*(\frac{x-x_c}{w})^2}$		$y = y_0 + A * e^{-\frac{1}{2}*(\frac{x-x_c}{w})^2}$	
Function	GaussAmp		GaussAmp	
Number of Points	63		63	
Number of Parameters	7		7	
F-test				
	F	Numer.DF	Denom.DF	Prob > F
	0.912	7	112	0.499
At the 0.05 significance level, the two datasets are not significantly different.				

Fit Reports												
			CTRL					INH				
	Peak	Parameter	Value	Standard Error	t-Value	Prob>|t|	Dependency	Value	Standard Error	t-Value	Prob>|t|	Dependency
Fit Parameters	Peak 1	y_0	0.759	0.955	0.795	0.429	0.562	1.932	0.855	2.258	0.027	0.522
		x_c	6.653	0.134	49.62	$5e^{-48}$	0.257	6.366	0.106	59.73	$2e^{-52}$	0.157
		w	2.174	0.258	8.423	$1e^{-11}$	0.866	1.685	0.170	9.859	$7e^{-14}$	0.781
		A	60.79	8.779	6.924	$4e^{-9}$	0.932	59.19	5.645	10.48	$8e^{-15}$	0.814
		FWHM	5.119	0.607	-	-	-	3.969	0.402	-	-	-
		Area	331.2	81.53	-	-	-	250.1	42.78	-	-	-
	Peak 2	y_0	0.759	0.955	0.795	0.429	0.562	1.932	0.855	2.258	0.027	0.522
		x_c	13.29	1.615	8.232	$3e^{-11}$	0.937	12.24	0.859	14.24	$2e^{-20}$	0.850
		w	5.411	1.150	4.701	$1e^{-5}$	0.916	5.156	0.656	7.849	$1e^{-10}$	0.825
		A	27.40	2.919	9.3886	$4e^{-13}$	0.753	30.50	2.290	13.32	$5e^{-19}$	0.632
		FWHM	12.74	2.710	-	-	-	12.14	1.546	-	-	-
		Area	371.7	104.1	-	-	-	394.3	63.31	-	-	-
Fit Statistics		Number of Points	63					63				
		Degrees of Freedom	56					56				
		Reduced Chi-Sqr	25.14					22.02				
		Residual Sum of Squares	1407					1233				
		R-Square (COD)	0.947					0.950				
		Adj. R-Square	0.942					0.945				

Statistics for comparison of Number of Major Branches (related to Suppl. Fig. 14e)

Fit Comparison of Data				
Description				
Input Data	CTRL<br>Source Data<br>TAB: Fig. 7d-g<br>Column: branchNodesPerBranch		INH<br>Source Data<br>TAB: Fig. 7d-g<br>Column: branchNodesPerBranch	
Fit Report	Fit Report CTRL (see bellow)		Fit Report INH (see bellow)	
Equation	$y = y_0 + A * e^{-\frac{1}{2}*(\frac{x-x_0}{w})^2}$		$y = y_0 + A * e^{-\frac{1}{2}*(\frac{x-x_0}{w})^2}$	
Function	GaussAmp		GaussAmp	
Number of Points	21		21	
Number of Parameters	7		7	
F-test				
	F	Numer.DF	Denom.DF	Prob > F
	0.2034	7	28	0.98199
At the 0.05 significance level, the two datasets are not significantly different.				

Fit Reports			CTRL					INH				
	Peak	Parameter	Value	Standard Error	t-Value	Prob>|t|	Dependency	Value	Standard Error	t-Value	Prob>|t|	Dependency
Fit Parameters	Peak 1	y0	0.936	2.203	0.424	0.677	0.570	1.401	1.866	0.750	0.465	0.546
		xc	7.757	0.661	11.72	1.26E-8	0.975	7.349	0.421	17.44	6.7E-11	0.944
		w	2.672	0.773	3.455	0.003	0.987	2.459	0.620	3.965	0.001	0.983
		A	156.3	180.8	0.864	0.401	0.999	137.2	117.6	1.165	0.263	0.998
		FWHM	6.293	1.821	-	-	-	5.791	1.460	-	-	-
		Area	1047	1508	-	-	-	845.8	933.8	-	-	-
	Peak 2	y0	0.936	2.203	0.424	0.677	0.570	1.401	1.866	0.750	0.465	0.546
		xc	11.95	7.249	1.648	0.121	0.998	11.41	3.675	3.104	0.007	0.997
		w	3.908	2.668	1.464	0.165	0.994	3.777	1.421	2.656	0.018	0.990
		A	80.35	103.9	0.772	0.452	0.998	101.5	64.76	1.567	0.139	0.997
		FWHM	9.204	6.283	-	-	-	8.896	3.348	-	-	-
		Area	787.3	1544.3	-	-	-	961.2	963.7	-	-	-
Fit Statistics		Number of Points	21					21				
		Degrees of Freedom	14					14				
		Reduced Chi-Sqr	43.74					33.21				
		Residual Sum of Squares	612.3					464.9				
		R-Square (COD)	0.993					0.994				
		Adj. R-Square	0.990					0.992				
