# Supplementary material for: Shared inflammatory glial cell signature after stab wound injury, revealed by spatial, temporal, and cell-type-specific profiling of the murine cerebral cortex
Source: Nat Commun. 2024 Apr 3;15:2866. doi: 10.1038/s41467-024-46625-w (PMC10991294; doi:10.1038/s41467-024-46625-w)
Supplement: Supplementary file 12 — Reporting Summary [file 41467_2024_46625_MOESM12_ESM.pdf]

nature portfolio

Corresponding author(s): Jovica Ninkovic

Last updated by author(s): Feb 15, 2024

Reporting Summary

Nature Portfolio wishes to improve the reproducibility of the work that we publish. This form provides structure for consistency and transparency in reporting. For further information on Nature Portfolio policies, see our Editorial Policies and the Editorial Policy Checklist.

Statistics

For all statistical analyses, confirm that the following items are present in the figure legend, table legend, main text, or Methods section.

n/a	Confirmed	
☐	☒	The exact sample size (n) for each experimental group/condition, given as a discrete number and unit of measurement
☐	☒	A statement on whether measurements were taken from distinct samples or whether the same sample was measured repeatedly
☐	☒	The statistical test(s) used AND whether they are one- or two-sided<br>*Only common tests should be described solely by name; describe more complex techniques in the Methods section.*
☒	☐	A description of all covariates tested
☐	☒	A description of any assumptions or corrections, such as tests of normality and adjustment for multiple comparisons
☐	☒	A full description of the statistical parameters including central tendency (e.g. means) or other basic estimates (e.g. regression coefficient) AND variation (e.g. standard deviation) or associated estimates of uncertainty (e.g. confidence intervals)
☐	☒	For null hypothesis testing, the test statistic (e.g. F, t, r) with confidence intervals, effect sizes, degrees of freedom and P value noted<br>*Give P values as exact values whenever suitable.*
☒	☐	For Bayesian analysis, information on the choice of priors and Markov chain Monte Carlo settings
☒	☐	For hierarchical and complex designs, identification of the appropriate level for tests and full reporting of outcomes
☒	☐	Estimates of effect sizes (e.g. Cohen's d, Pearson's r), indicating how they were calculated

Our web collection on statistics for biologists contains articles on many of the points above.

Software and code

Policy information about availability of computer code

Data collection	Leica TCS SP8 & LASX software (3.5.6.21594), Carl Zeiss Axio Imager.M2m & Zen software (v2.3), Illumina HiSeq1500, Illumina HiSeq 4000, Illumina NovaSeq6000
Data analysis	Confocal images were analyzed with ImageJ (v2.1.0/1.53f). Microglia morphology parameters were determined using MATLAB (9.4.0.813654) and the publicly deposited MATLAB script of Heindl et al. 2018 (https://github.com/isdneuroimaging/mmqt). Downsampling of control microglia for microglia morphology analysis was conducted using the tool Data Sampler in Orange (v.3.32.0). Statistical analysis was performed using GraphPad Prism (v9.3.1). Demultiplexing of stRNA-seq data was performed using Je tool (v1.2) and for alignment Space Ranger (v.1.2.2) was used. Primary processing of raw scRNA-seq sequencing data to fastq files was carried out with Illumina's bcl2fastq (v2.20.0.422) and alignment was performed using Cell Ranger (v3.0.2 or v6.0.0). R(v4.0.2) package scran was used for scRNA-seq normalization. Python(v3.8.8) packages Scanpy(v1.7.1) and Squidpy(v1.2.2) were used to analyze sc- & st-RNA-seq datasets. Detailed analysis pipelines are deposited elsewhere (https://github.com/NinkovicLab/Koupourtidou-Schwarz-et-al). For the comparisons of the overlapping genes and the gene ontology analysis the R(v4.3.1) package UpSetR(v1.4.0) and clusterProfile(v4.8.2) were used respectively, whereas networkD3(v0.4) was used for the Sankey Diagram. Microglia morphology analysis was done using OriginPro (version 2021b).

For manuscripts utilizing custom algorithms or software that are central to the research but not yet described in published literature, software must be made available to editors and reviewers. We strongly encourage code deposition in a community repository (e.g. GitHub). See the Nature Portfolio guidelines for submitting code & software for further information.

Data

Policy information about availability of data

All manuscripts must include a data availability statement. This statement should provide the following information, where applicable:

- Accession codes, unique identifiers, or web links for publicly available datasets
- A description of any restrictions on data availability
- For clinical datasets or third party data, please ensure that the statement adheres to our policy

All sequencing data generated in association with this study are available in the Gene Expression Omnibus as a SuperSeries under accession number GSE226211 [https://www.ncbi.nlm.nih.gov/geo/query/acc.cgi?acc=GSE226211]. scRNA-seq data are available under accession number GSE226207 [https://www.ncbi.nlm.nih.gov/geo/query/acc.cgi?acc=GSE226207] and spatial transcriptomic under GSE226208 [https://www.ncbi.nlm.nih.gov/geo/query/acc.cgi?acc=GSE226208]. The mouse reference genome mm10 [https://www.10xgenomics.com/support/software/cell-ranger/downloads/cr-ref-build-steps] was used for the data alignment. The online available databases for the mouse brain [http://mousebrain.org/adolescent/genesearch.html] and the immune cells [http://rstats.immgen.org/MyGeneSet_New/index.html] were used for the cluster annotation of the scRNA-seq data.

scRNA-seq data from other studies referenced in Fig. 5 and Supplementary Fig. 9 are available from the GEO with accession number GSE180862 [https://www.ncbi.nlm.nih.gov/geo/query/acc.cgi?acc=GSE180862] and GSE174574 [https://www.ncbi.nlm.nih.gov/geo/query/acc.cgi?acc=GSE174574], respectively. The plots in Supplementary Fig. 9f-g were generated from the online searchable database GliaSeq containing the scRNA-seq analysis by Hasel et al. [https://liddelowlab.shinyapps.io/GliaSeqPro/].

Source data are provided with this paper.

Research involving human participants, their data, or biological material

Policy information about studies with human participants or human data. See also policy information about sex, gender (identity/presentation), and sexual orientation and race, ethnicity and racism.

Reporting on sex and gender	n/a
Reporting on race, ethnicity, or other socially relevant groupings	n/a
Population characteristics	n/a
Recruitment	n/a
Ethics oversight	n/a

Note that full information on the approval of the study protocol must also be provided in the manuscript.

Field-specific reporting

Please select the one below that is the best fit for your research. If you are not sure, read the appropriate sections before making your selection.

[x] Life sciences [ ] Behavioural & social sciences [ ] Ecological, evolutionary & environmental sciences

For a reference copy of the document with all sections, see nature.com/documents/nr-reporting-summary-flat.pdf

Life sciences study design

All studies must disclose on these points even when the disclosure is negative.

Sample size	No statistical methods were used to pre-determine samples sizes. Sample sizes were selected based on previous reports for similar experiments. We followed the sample size used in the papers using the same readouts for addressing reactivity of glial cells: Ohlig et al., 2021; Canhos et al., 2021; Frik et al., 2018; Sirko et al., 2013.
Data exclusions	Number of control microglia used for morphological analysis was randomly downsampled to obtain similar number of inhibitor-treated microglia for direct comparison.
Replication	st-RNA-seq experiment as well as experiments to visualize injury-induced cluster VI genes were performed once. st-RNA-seq experiments were done on two different sections showing the same expression pattern. Moreover, the RNAScope experiments (visualization of cluster VI) are independent validations of the stRNA-seq. As we could confirm all st-RNA-seq based expression patterns, in our opinion there is no need for additional st-RNA-seq experiments.<br>sc-RNA-seq experiments were performed in at least 2 replicates. Visualization of the shared inflammatory genes was repeated twice. Histological determination of astrocyte and oligodendrocyte proliferation in control- and inhibitor-treated animals was repeated twice. Experiments to assess morphological microglia features were conducted in two independent experimental rounds. Astrocyte reacitvity at 5 dpi was performed once (GFAP coverage, GFAP/NGAL+ cell) or twice (NGAL intensity). Microglia morphology analysis was performed in two independent experimental rounds.<br>All attempts of replication were successful.

Randomization	Mice were selected randomly and allocated into different experimental groups. No additional randomization was used during data collection.
Blinding	Investigators were blinded while acquiring and analyzing data from different experimental groups. Once the analysis was completed, the group allocation was revealed.

Reporting for specific materials, systems and methods

We require information from authors about some types of materials, experimental systems and methods used in many studies. Here, indicate whether each material, system or method listed is relevant to your study. If you are not sure if a list item applies to your research, read the appropriate section before selecting a response.

Materials & experimental systems

n/a	Involved in the study
☐	☒ Antibodies
☒	☐ Eukaryotic cell lines
☒	☐ Palaeontology and archaeology
☐	☒ Animals and other organisms
☒	☐ Clinical data
☒	☐ Dual use research of concern
☒	☐ Plants

Methods

n/a	Involved in the study
☒	☐ ChIP-seq
☒	☐ Flow cytometry
☒	☐ MRI-based neuroimaging

Antibodies

Antibodies used

1.) anti-GFP, Aves Lab, cat. number: GFP-1020, lot number: GFP3717982, chicken polyclonal, dilution 1:500
2.) anti-GFAP, Abcam, cat. number: ab53554, lot number: GR3418475-1, goat polyclonal, dilution 1:300
3.) anti-GFAP, Sigma-Aldrich, cat. number: G3893, lot number: 0000167864, mouse monoclonal IgG1, Clone: G-A-5 clone, dilution 1:500
4.) anti-IBA1, Wako, cat. number: 019-19741, lot number: SKP3626, rabbit polyclonal, dilution 1:500 and 1:200 for microglia morphology
5.) anti-NGAL, Thermofisher, cat. number: PA5-79590, lot number: WI3386575, rabbit polyclonal, dilution 1:500
6.) anti-SERPINA3N, R&D Systems, cat. number: AF4709-SP, lot number: CBKW0321031, goat polyclonal, dilution 1:500
7.) anti-CD68, BioRad, cat. number: MCA1957T, lot number: 153583, rat monoclonal, dilution 1:600
8.) anti-OLIG2, Millipore cat. number: MABN50, lot number: 3709426, mouse monoclonal IgG2aκ, Clone: 211F1.1, dilution 1:200
9.) anti-P2Y12, AnaSpec, cat. number: AS-55043A, lot number: TB0501, rabbit polyclonal, diluation 1:750

i.) donkey anti-chick IgY Alexa Fluor 488, Dianova, cat. number: 703-545-155, lot number: 2304258, dilution 1:1000
ii.) donkey anti-goat IgG Alexa Fluor 647, Jackson Immuno Research, cat. number: 705-605-003, lot number: 153846, dilution 1:1000
iii.) goat anti-mouse IgG1 Alexa Fluor 546, ThermoFisher, cat. number: A-21123, lot number: 2228626, dilution 1:1000
iv.) goat anti-rabbit IgG Alexa Fluor 546, ThermoFisher, cat. number: A-11010, lot number: 2291627, dilution 1:1000
v.) goat anti-rabbit IgG Alexa Fluor 633, ThermoFisher, cat. number: A-21070, lot number: 1889306, dilution 1:1000
vi.) goat anti-rat IgG Alexa Fluor 488, ThermoFisher, cat. number: A-11006, lot number: 2005935, dilution 1:1000
vii.) goat anti-mouse IgG2a Alexa Fluor 488, ThermoFisher, cat. number: A-21131, lot number: 2136788, dilution 1:1000
viii.) goat anti-mouse IgG1 Alexa Flour 488, ThermoFisher, cat. number: A-21121, lot number: 2339820, dilution 1:1000
ix.) goat anti-rat Alexa Flour 546, ThermoFisher, cat. number: A-11081, lot number: 2304272, dilution 1:1000

Validation

1.) anti-GFP antibody has been validated previously in mouse brain tissue (Canhos et al. 2020, PMID: 32744730)
2.) anti-GFAP antibody (goat) has been validated previously in mouse brain tissue (Konishi et al. 2020, PMID: 32959911)
3.) anti-GFAP antibody (mouse IgG1) has been validated previously in mouse brain tissue (Ohlig et al. 2021, PMID: 34549820)
4.) anti-IBA1 antibody has been validated previously in mouse brain tissue (Heindl et al. 2018, PMID: 29725290)
5.) anti-NGAL antibody has been validated previously in mouse kidney tissue (Klinkhammer et al. 2020, PMID: 32086278)
6.) anti-SERPINA3N antibody has been validated previously in mouse brain tissue (Kaya et al. 2022, PMID: 36280798)
7.) anti-CD68 antibody has been validated previously in mouse spinal cord tissue (Li et al. 2020, PMID: 33029008)
8.) anti-OLIG2 antibody has been validated previously in mouse brain tissue (Ohlig et al. 2021, PMID: 34549820)
9.) anti-P2Y12 antibody has been validated previously in mouse brain tissue (Bernier et al. 2019, PMID: 31167136)

Animals and other research organisms

Policy information about studies involving animals; ARRIVE guidelines recommended for reporting animal research, and Sex and Gender in Research

Laboratory animals

Species: Mus musculus, strains: C57Bl6/J (strain #000664), NG2-CreERT2xCAG-GFP (CAG-GFP strain #024636), NG2-EYFP, sex: male, age: 8-12 weeks. Mice were housed under the German and European guidelines for the use of animals for research purposes. With room temperature maintained within the range 20–22 °C, relative humidity ranged between 45–55%, light cycle adjusted to 12 h light:12 h dark period and free access to water and food.

Wild animals	No wild animals were used in this study
Reporting on sex	Given the previous reported sexual dimorphism in glial cell responses to pathologies (see e.g. Villapol et al. 2017), we decided to exclusively focus our study on one sex to ensure data consistency and enable a targeted analysis within our predefined research scope. Hence, only male mice were used in this study.
Field-collected samples	No field-collected samples were used for this study.
Ethics oversight	All experiments carried out in this study have been approved and overseen by the government of Upper Bavaria (animal license number: ROB-55.2-2532.Vet_02-20-158) and the Saarland state's ''Landesamt für Verbraucherschutz" in Saarbrücken/Germany (animal license number: 17/2023).

Note that full information on the approval of the study protocol must also be provided in the manuscript.

Plants

Seed stocks	*Report on the source of all seed stocks or other plant material used. If applicable, state the seed stock centre and catalogue number. If plant specimens were collected from the field, describe the collection location, date and sampling procedures.*
Novel plant genotypes	*Describe the methods by which all novel plant genotypes were produced. This includes those generated by transgenic approaches, gene editing, chemical/radiation-based mutagenesis and hybridization. For transgenic lines, describe the transformation method, the number of independent lines analyzed and the generation upon which experiments were performed. For gene-edited lines, describe the editor used, the endogenous sequence targeted for editing, the targeting guide RNA sequence (if applicable) and how the editor was applied.*
Authentication	*Describe any authentication procedures for each seed stock used or novel genotype generated. Describe any experiments used to assess the effect of a mutation and, where applicable, how potential secondary effects (e.g. second site T-DNA insertions, mosiacism, off-target gene editing) were examined.*
